# OLIG2 regulates lncRNAs and its own expression during oligodendrocyte lineage formation

**DOI:** 10.1186/s12915-021-01057-6

**Published:** 2021-06-25

**Authors:** Haichao Wei, Xiaomin Dong, Yanan You, Bo Hai, Raquel Cuevas-Diaz Duran, Xizi Wu, Natasha Kharas, Jia Qian Wu

**Affiliations:** 1 The Vivian L. Smith Department of Neurosurgery, McGovern Medical School, The University of Texas Health Science Center at Houston, Houston, TX USA; 2 Center for Stem Cell and Regenerative Medicine, UT Brown Foundation Institute of Molecular Medicine, Houston, TX USA; 3 Tecnologico de Monterrey, Escuela de Medicina y Ciencias de la Salud, Monterrey, N.L. Mexico; 4 Department of Neurobiology and Anatomy, The University of Texas Medical School at Houston, Houston, TX USA; 5 MD Anderson Cancer Center UTHealth Graduate School of Biomedical Sciences, Houston, TX USA

**Keywords:** Oligodendrocyte development, OLIG2, LncRNAs, Histone modification, Transcriptional regulation, Regulation after transcription

## Abstract

**Background:**

Oligodendrocytes, responsible for axon ensheathment, are critical for central nervous system (CNS) development, function, and diseases. OLIG2 is an important transcription factor (TF) that acts during oligodendrocyte development and performs distinct functions at different stages. Previous studies have shown that lncRNAs (long non-coding RNAs; > 200 bp) have important functions during oligodendrocyte development, but their roles have not been systematically characterized and their regulation is not yet clear.

**Results:**

We performed an integrated study of genome-wide OLIG2 binding and the epigenetic modification status of both coding and non-coding genes during three stages of oligodendrocyte differentiation in vivo: neural stem cells (NSCs), oligodendrocyte progenitor cells (OPCs), and newly formed oligodendrocytes (NFOs). We found that 613 lncRNAs have OLIG2 binding sites and are expressed in at least one cell type, which can potentially be activated or repressed by OLIG2. Forty-eight of them have increased expression in oligodendrocyte lineage cells. Predicting lncRNA functions by using a “guilt-by-association” approach revealed that the functions of these 48 lncRNAs were enriched in “oligodendrocyte development and differentiation.” Additionally, bivalent genes are known to play essential roles during embryonic stem cell differentiation. We identified bivalent genes in NSCs, OPCs, and NFOs and found that some bivalent genes bound by OLIG2 are dynamically regulated during oligodendrocyte development. Importantly, we unveiled a previously unknown mechanism that, in addition to transcriptional regulation via DNA binding, OLIG2 could self-regulate through the 3′ UTR of its own mRNA.

**Conclusions:**

Our studies have revealed the missing links in the mechanisms regulating oligodendrocyte development at the transcriptional level and after transcription. The results of our research have improved the understanding of fundamental cell fate decisions during oligodendrocyte lineage formation, which can enable insights into demyelination diseases and regenerative medicine.

**Supplementary Information:**

The online version contains supplementary material available at 10.1186/s12915-021-01057-6.

## Background

The mammalian central nervous system (CNS) is made up of diverse cell types that belong to distinct functional lineages and are developmentally specified by transcription factors (TFs) [1, 2]. Oligodendrocyte progenitor cells (OPCs) differentiate from neural stem cells (NSCs) and can give rise to mature oligodendrocytes responsible for myelination. Axon ensheathment is critical for CNS development, function, and diseases such as multiple sclerosis (MS), leukodystrophies, stroke, and spinal cord or brain injury [3–7]. However, the molecular mechanisms regulating oligodendrocyte formation and differentiation are not yet fully understood.

A number of stage-specific TFs have been reported to control oligodendrocyte specification, differentiation, and myelination [8]. The basic-helix-loop-helix (bHLH) transcription factor oligodendrocyte TF 1/2 (OLIG1/2) are master regulators of oligodendrocyte development [9–12]. *Olig1* and *Olig2* have potentially redundant but also non-overlapping roles in the proliferation and differentiation of neural progenitors [13]. Previous studies reported that ablation of *Olig1* had an impact on the later stage of oligodendrocyte development [12, 14] and knocking out *Olig2* in mice led to the failure of oligodendroglial lineage generation [12]. Specific deletion of *Olig2* in OPCs caused hypomyelination by reducing oligodendrocyte differentiation, while the ablation of *Olig2* in immature oligodendrocyte induced maturation of oligodendrocytes, myelination, and remyelination in mice [15]. Moreover, overexpression of *Olig2* in NSCs promoted the generation of OPCs and mature oligodendrocytes both in vitro and in vivo [11, 16].

Previous studies have shown that OLIG2 may activate or repress gene expression directly or cooperatively with other factors [17–19]. OLIG2 regulates many important protein-coding genes during oligodendrocyte formation (e.g., *Sox10*) [20]. Additionally, previous studies from our group and others have demonstrated that OLIG2 also controls many long non-coding RNAs (lncRNAs) [21, 22]. lncRNAs (> 200 bp) play crucial roles in transcriptional regulation during various biological processes including embryonic stem cells (ESCs) differentiation, neurogenesis, and cancer [23, 24].

It is of critical importance to investigate lncRNAs, because only less than 2% of the genome is associated with protein-coding genes, and a large number of disease-causing mutations reside in the non-coding regions [25]. Several studies have revealed that lncRNAs are transcribed in developmental- and tissue-specific manners and regulate cell fate determination [26–30]. For example, the depletion of dozens of lincRNAs (long intergenic noncoding RNAs) causes ESCs to exit the pluripotent state or increase lineage commitment [31]. Also, multiple mouse knockout models have proven that lncRNAs are required for brain development in vivo [28]. Also, lncRNAs are regulated by cell-type-specific TFs, such as the pluripotency TFs SOX2, OCT4, and NANOG in ESCs, or the RE1 silencing TF (REST) during neuronal differentiation [31–33]. In our previous studies, we found that *lnc-OPC* was highly expressed in OPCs and repressed by OLIG2 [21, 34]. The deletion of *lnc-OPC* significantly reduced OPCs formation and affected the expression of genes associated with differentiation from NSCs to OPCs [21]. LncRNAs often bind to ubiquitous regulatory proteins such as chromatin modification complexes to guide them to specific locations in the genome, controlling a number of downstream genes to define cell-type-specific expression programs. Such a mechanism resolves the paradox that a small repertoire of chromatin-modifying complexes, which often have RNA-binding domains but little DNA sequence specificity, can establish complex epigenetic states across different cell types arising throughout development [35, 36]. For example, He et al. have demonstrated that *lncOL1* and SUZ12/PRC2 could form a complex to promote oligodendrocyte differentiation [22].

Evidence shows that dynamic epigenetic modification plays an essential role during the process of oligodendrocyte differentiation [37–39]. In particular, bivalent domains containing both repressive H3K27m3 (K27) and active H3K4m3 (K4) marks reportedly play important roles in pluripotency and cell differentiation [40]. In ESCs, genes with bivalent domains in the promoter (bivalent genes) were silent yet poised for activation upon differentiation [40]. However, little is known about the function of such bivalent genes during the process of oligodendrocyte formation and differentiation or about how they are regulated.

In addition to regulation at transcription level, regulation after transcription involves RNA processing (such as alternative splicing, capping, and polyadenylation), RNA editing, mRNA stability, and translation. Previous studies have demonstrated that certain genes could regulate their expression through protein interaction with their own mRNAs. For example, TDP-43 and huntingtin protein (HTT) in the CNS [41, 42]. However, it is not yet known whether OLIG2 has such a mechanism of regulation.

In previous studies, we identified the cell-type-specific gene markers by analyzing NSCs, OPCs, and newly formed oligodendrocytes (NFOs) transcriptomes [43]. To further investigate the mechanisms of regulation during oligodendrocyte differentiation, we have performed ChIP-Seq (chromatin immunoprecipitation sequencing) experiments by in vivo purifying oligodendrocyte lineage cells of different stages of oligodendrocyte development using mouse models. We integrated ChIP-Seq data for OLIG2 and epigenetic status together with RNA-Seq (RNA sequencing) data from NSCs, OPCs, and NFOs. In the current study, we have identified the protein-coding and lncRNA genes bound by OLIG2 in vivo in NSCs, OPCs, and NFOs. Motif analysis showed that binding motifs for other TFs were also found in the OLIG2 binding region, likely cooperating with OLIG2 in regulating oligodendrocyte development. We found that lncRNAs can be either activated or repressed by OLIG2, potentially functioning during oligodendrocyte formation and differentiation. These OLIG2-bound lncRNAs were functionally enriched in ‘oligodendrocyte differentiation’ based on “guilt-by-association” analysis. Based on ChIP-Seq analysis of H3K27m3 (K27) and H3K4m3 (K4) marks, we identified the bivalent genes in NSC, OPC, and NFO stages. These bivalent genes were dynamically regulated during oligodendrocyte differentiation and some of them were bound by OLIG2. Intriguingly, we found AU-rich elements (AREs) in the 3′ UTR (untranslated region) of *Olig2* mRNAs and demonstrated for the first time that OLIG2 can act not only as a transcriptional factor by binding to genomic DNA but also be involved in the regulation of *Olig2* after transcription via the 3′ UTR of its own mRNA.

## Results

### Functional enrichment of OLIG2-binding genes at oligodendrocyte formation and differentiation stages

**Fig. 1:**
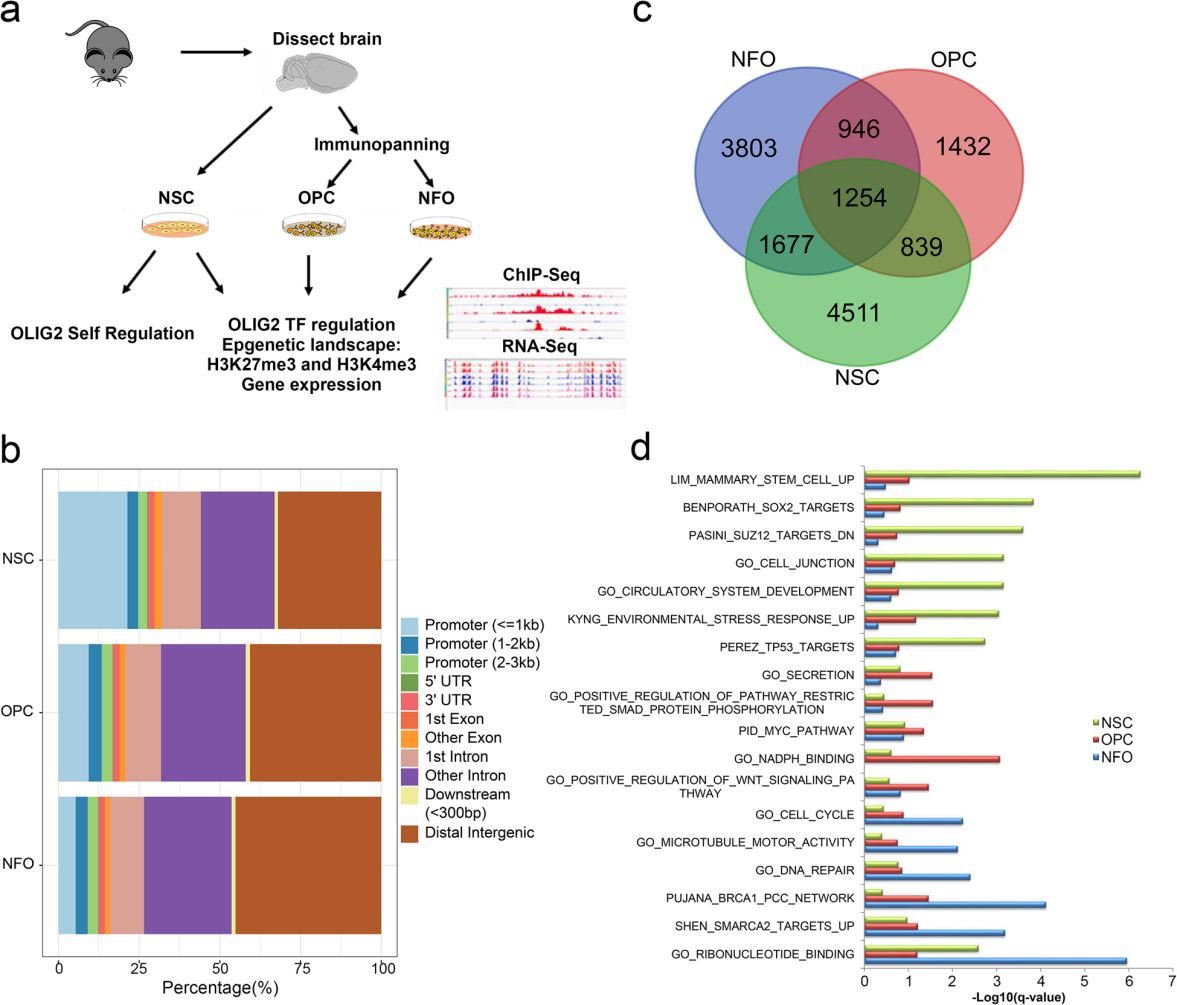
Experimental scheme and the genome-wide binding of OLIG2 in NSCs, OPCs, and NFOs. **a** Experimental scheme and data integration. **b** Genomic distribution of OLIG2 called peaks in NSCs, OPCs, and NFOs. **c** Overlapping OLIG2 binding genes in NSCs, OPCs, and NFOs. **d** Functional enrichment of specific genes in NSCs, OPCs, and NFOs

Figure 1a shows the overall experimental scheme and data integration. To investigate the target genes of OLIG2 during the NSC-OPC-NFO differentiation process, we have purified oligodendrocyte lineage cells of different stages of oligodendrocytes development using mouse models by immunopanning method in vivo [44]. Subsequently, we performed OLIG2 ChIP-Seq experiments and bioinformatics analysis in NSCs, OPCs, and NFOs. The same pipeline and consistent parameters were used to analyze OLIG2 called peaks for uniformity and comparability across cell types (see the “Methods” section). Using the SPP program from the ENCODE project, we identified 13,959, 7384, and 16,911 OLIG2 called peaks in NSCs, OPCs, and NFOs, respectively. The distribution of OLIG2 called peaks in the genome showed that 32%, 40.64%, and 45.15% of them were localized in distal intergenic regions in NSCs, OPCs, and NFOs, respectively, and that 21.35%, 9.36%, and 5.34% were localized close to promoters (< 1 Kb from transcription start sites (TSS)) in NSCs, OPCs, and NFOs, respectively (Fig. 1b). Totals of 10,161, 4482, and 9030 peaks were identified within 5 kb upstream of the TSS and the gene body in NSCs, OPCs, and NFOs (Additional file 1: Table S1). We annotated OLIG2 ChIP-Seq called peaks and identified OLIG2 target genes including 8281 genes in NSCs (5539 protein-coding genes and 2742 lncRNAs), 4471 genes in OPCs (3233 protein-coding genes and 1238 lncRNAs), and 7680 genes in NFOs (5388 protein-coding genes and 2292 lncRNAs) (Additional file 1: Table S1). We annotated called peaks to genes and performed a functional enrichment using MSigDB terms. Results indicated that 3803 genes with called peaks only in NSCs were enriched in stem cell terms, and SOX2- and SUZ12-target genes; 1432 genes with called peaks only in OPCs were enriched in NADPH, WNT, and MYC pathways; and 4511 genes with called peaks only in NFOs were enriched in cell cycle, microtubule, and ribonucleotide binding terms (Fig. 1c, d).

### OLIG2 can function either as a repressor or an activator in regulating oligodendrocyte formation and differentiation

**Fig. 2:**
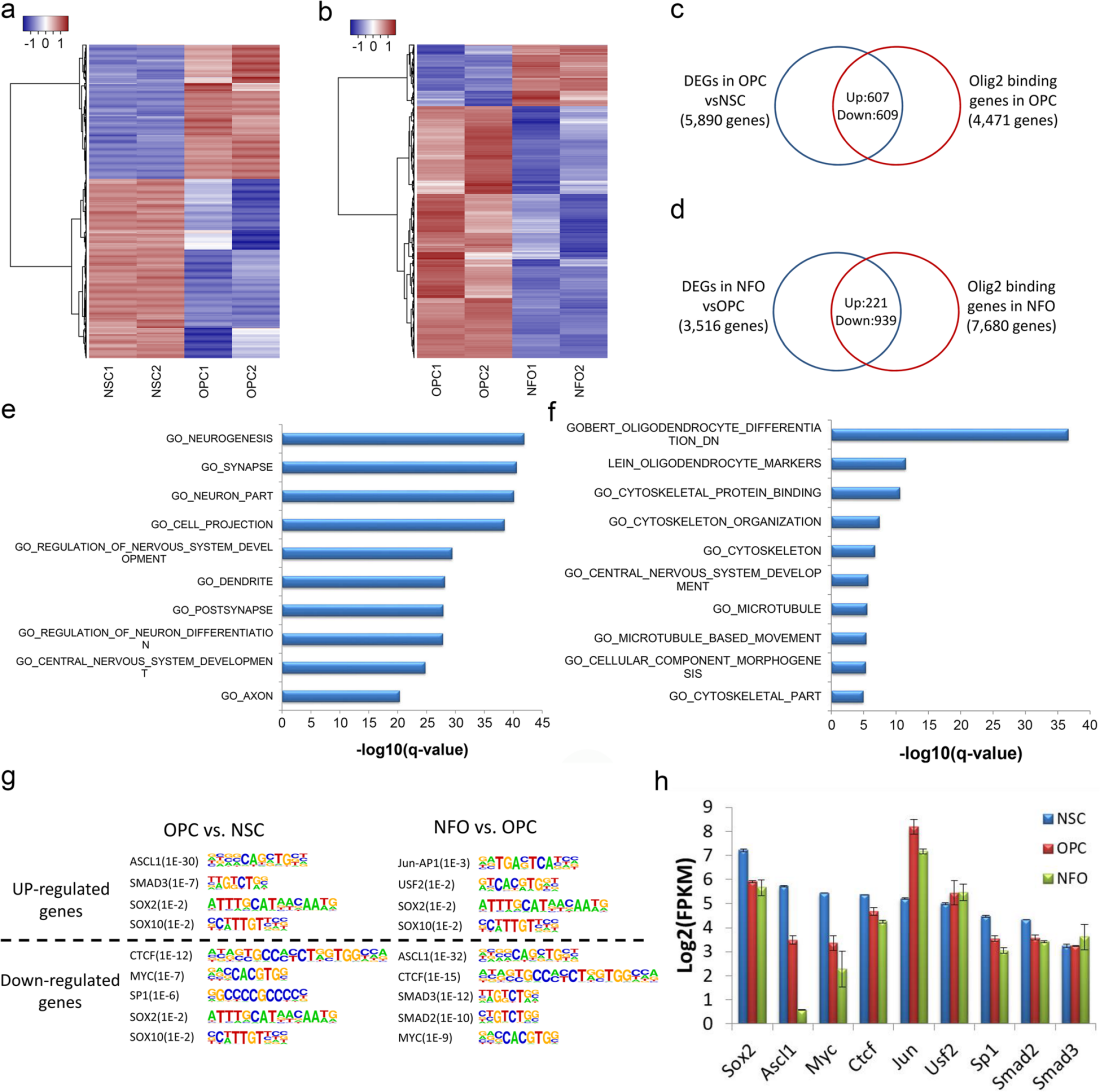
OLIG2 activates or represses gene expression during transitions from NSCs to OPCs and OPCs to NFOs. **a**, **b** Heatmaps of DEGs in OPCs compared with NSCs (**a**) and NFOs compared with OPCs (**b**). **c**, **d** Overlap of DEGs bound by OLIG2 in OPCs compared with NSCs (**c**) and NFOs compared with OPCs (**d**). **e**, **f** Gene set enrichment of downregulated (**e**) or upregulated (**f**) genes that are also bound by OLIG2 in NFOs compared with OPCs. **g** Some examples of top binding motifs of TFs that are enriched in the OLIG2 called peak regions. **h** Gene expression of TFs

To identify the genes regulated by OLIG2 during oligodendrocyte development, we correlated the ChIP-Seq data for OLIG2 and the RNA-Seq data from purified NSCs, OPCs, and NFOs. In our previous work, we studied the transcriptomes of NSCs from embryonic (E14.5) CD-1 mouse brain cortex [21] and OPCs and NFOs purified by immunopanning from mouse cerebral cortices (P17) [43]. Firstly, we detected differentially expressed genes (DEGs) during oligodendrocyte formation in the transition from NSCs to OPCs and during oligodendrocyte differentiation in the transition from OPCs to NFOs (Fig. 2a, b, Additional file 2: Table S2). Comparing OPCs to NSCs, 5890 significant DEGs were observed and 1216 of them (607 upregulated genes and 609 downregulated genes) were bound by OLIG2 in OPCs as shown by ChIP-Seq (Fig. 2a, c). Another 3516 DEGs were obtained by comparing NFOs to OPCs. A total of 1160 of these (221 upregulated genes and 939 downregulated genes) were bound by OLIG2 in NFOs (Fig. 2b, d). The genes that were down-regulated or repressed during the transition from OPCs to NFOs were strongly enriched in the terms “nervous system development,” “neuron part,” and “synapse” (Fig. 2e), and the upregulated or activated genes were enriched in “oligodendrocyte differentiation” and “cytoskeletal protein” (Fig. 2f).

The above analysis implies that OLIG2 binding did not always lead to gene activation but also to gene repression, which raises a question as to whether this could due to the recruitment of other TFs. We obtained DEGs with OLIG2 called peaks in OPCs or NFOs and analyzed the TF motifs using Homer known motifs analysis among these peaks (Fig. 2g). For example, ASCL1 and SMAD3 motifs were highly enriched in both OPCs vs. NSCs up-regulated genes and NFOs vs. OPCs down-regulated genes; SMAD2 motifs were highly enriched in NFOs vs. OPCs down-regulated genes; MYC motifs was enriched in OPCs vs. NSCs and NFOs vs. OPCs downregulated genes. In addition to these TF motifs, we also found that CTCF motifs were enriched in downregulated genes in OPCs vs. NSCs and NFOs vs. OPCs; Jun-AP1 and USF2 motifs are enriched in NFOs vs. OPCs upregulated genes. Motifs for SOX family proteins were enriched in both OPCs vs. NSCs up- and downregulated genes and NFOs vs. OPCs upregulated genes. Because of fewer upregulated genes in NFOs vs. OPCs with OLIG2 called peaks, the statistics of TF motifs among these genes is less significant compared to other stages. RNA-Seq data showed that these TFs were indeed expressed in NSCs, OPCs, and NFOs (Fig. 2h).

### OLIG2 regulates lncRNAs during oligodendrocyte development

**Fig. 3:**
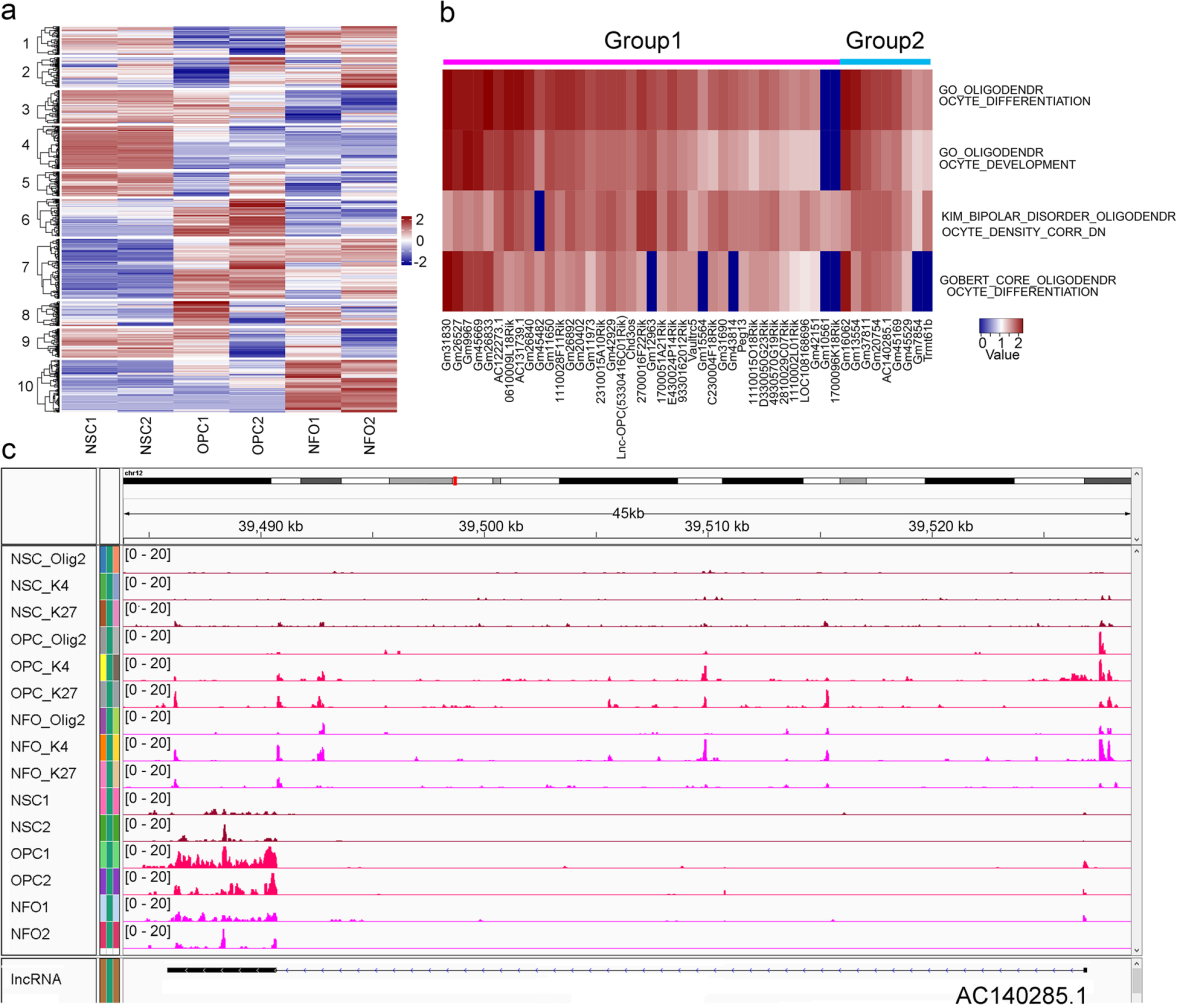
LncRNAs bound by OLIG2 in NSCs, OPCs, and NFOs. **a** *K*-means clustering of lncRNAs identified in NSCs, OPCs, and NFOs. **b** The potential function of 48 lncRNAs in cluster 7. Group1 genes (39 lncRNAs) have OLIG2 called peaks in NSCs but not in OPCs or NFOs; group 2 genes (9 lncRNAs) have OLIG2 called peaks in OPCs and NFOs but not in NSCs. Color depth represents NES (normalized enrichment score) calculated by GSEA and indicates association strength. **c** ChIP-Seq and gene expression of *AC140285.1* in NSCs, OPCs, and NFOs

Previously, we have demonstrated the functional role of lncRNAs in oligodendrocyte formation [21]. We also showed that OLIG2 could regulate the expression of *lnc-OPC* (*5330416C01Rik*) by acting as a transcriptional repressor [21]. To comprehensively identify OLIG2-bound lncRNAs during oligodendrocyte differentiation, we focused on the lncRNAs that have OLIG2 called peaks and FPKM (Fragments Per Kilobase of transcript per Million mapped reads) > 1 in at least one cell type. A total of 613 lncRNAs were retained for analysis of gene expression patterns and 10 expression patterns were identified using the k-means clustering method (Fig. 3a, Additional file 3: Table S3). Among genes in cluster 3, there were 56 lncRNAs with low expression in NFOs and high expression in NSCs and OPCs, including four genes (*Mir99ahg*, *2610316D01Rik*, *Pantr1*, and *B230311B06Rik*) with OLIG2 called peaks in NSCs and OPCs but not in NFOs, and seven genes with OLIG2 called peaks in NFOs but not in NSCs or OPCs. The genes in cluster 4 (73 lncRNAs) and cluster 5 (42 lncRNAs) exhibited high expression in NSCs and low expression in OPCs and NFOs (e.g., *Crnde*). A total of 45 genes have OLIG2 called peaks only in NSCs but not in OPCs or NFOs and five genes were annotated with OLIG2 called peaks in OPCs and NFOs but not in NSCs. In cluster 6, 63 lncRNAs including *Sox2ot* had high expression in OPCs but not in NSCs or NFOs. Four genes were annotated with OLIG2 called peaks only in OPCs, and nine genes had called peaks only in NSCs and NFOs. A total of 88 lncRNAs are grouped into cluster 10 with low expression in NSCs and OPCs, but high expression in NFOs, including four genes with OLIG2 called peaks only in NSCs and OPCs and 17 genes only in NFOs.

The genes in cluster 7 (101 lncRNAs) were expressed at lower levels in NSCs and higher levels in OPCs and NFOs. A total of 39 genes (including *lnc-OPC*, group 1) were annotated with OLIG2 called peaks only in NSCs but not in OPCs and NFOs, and nine lncRNAs (group 2) had OLIG2 called peaks in OPCs and NFOs but not in NSCs (Fig. 3b). We predicted the potential roles of these lncRNAs using a “guilt-by-association” analysis. First, we computed the gene expression Pearson correlations between each lncRNA and protein-coding genes and then ranked protein-coding genes by their correlation coefficients [45]. Significantly enriched gene sets were identified using the Gene Set Enrichment Analysis (GSEA) [46, 47]. The functions of these 48 lncRNAs were enriched for the term “oligodendrocyte differentiation” (Fig. 3b). For example, *AC140285.1* has lower expression in NSCs but higher expression in OPCs and NFOs according to RNA-Seq and qPCR validation (Fig. 3c and Additional file 4: Fig S1) from NSCs, OPCs, and NFOs. Moreover, OLIG2 ChIP-Seq results showed that this gene is annotated with OLIG2 called peaks in OPCs and NFOs but not in NSCs (Fig. 3c). These correlation results of OLIG2 ChIP-Seq with RNA-Seq in NSCs, OPCs, and NFOs suggested that *AC140285.1* might be activated by OLIG2 in OPCs and NFOs.

### Epigenetic landscape: genomic distributions of H3K4me3 (K4) and H3K27me3 (K27) marks during the course of oligodendrocyte development

**Fig. 4:**
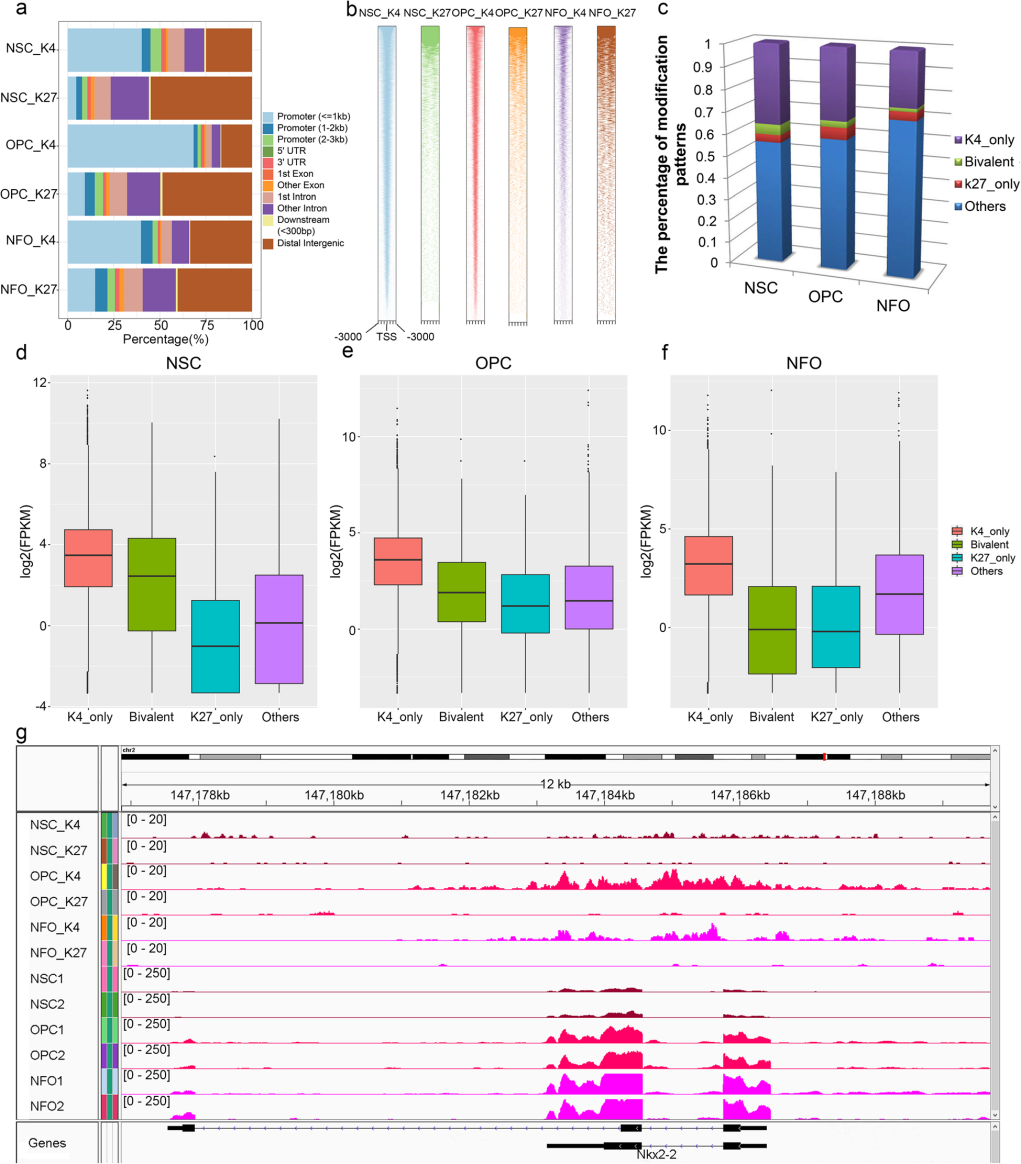
Distribution of H3K4me3 (K4) and H3K27me3 (K27) in NSCs, OPCs, and NFOs. **a** Genomic distribution of H3K4me3 and H3K27me3 called peaks in NSCs, OPCs, and NFOs. **b** Heatmap of H3K4me3 (K4) and H3K27me3 (K27) ChIP-Seq data in NSCs, OPCs, and NFOs in the interval from − 3 kb to + 3 kb from the TSS. **c** The percentage of genes in NSCs, OPCs, and NFOs associated with only H3K4me3, bivalent marks, only H3K27me3, or other states. **d**–**f**. Box plot of expression values of genes from different epigenomic categories in NSCs (**d**), OPCs (**e**), and NFOs (**f**). **g** Profiles of H3K4me3 and H3K27me3 marks and RNA-Seq visualized using IGV (*Nkx2-2*)

To examine the dynamic epigenetic modification during NSCs differentiation into OPCs and NFOs, we performed ChIP-Seq experiments to identify H3K4me3 (activated chromatin marks) and H3K27me3 (repressed chromatin marks) in NSCs, OPCs, and NFOs, followed by bioinformatics analysis [48]. The majority (40.6%, 68.28%, and 39.76% in NSCs, OPCs, and NFOs, respectively) of H3K4me3 called peaks was distributed in proximal promoter regions (≤ 1 kb from TSS), while the majority (54.96%, 48.6%, and 40.35% in NSCs, OPCs, and NFOs, respectively) of H3K27me3 called peaks were found in distal intergenic regions (Fig. 4a). The densities of read count frequencies for H3K4me3 and H3K27me3 revealed that H3K4me3 called peaks were enriched close to the TSS and that H3K27me3 called peaks were enriched in regions distal to the TSS (Fig. 4b).

We also constructed a state-of-the-art regulation database using WashU EpiGenome Browser [49] that provides an interactive, searchable interface for querying and exploring OLIG2 and epigenetic regulation across the stages during oligodendrocyte development. The database is a CGI application and is publicly available to the research community (http://jiaqianwulab.org/OLIG2chip/oligodendrocyte_chip.html).

To identify associations between epigenetic modification and gene expression, we annotated total peaks identified (see Methods). We annotated the peaks within 2 kb upstream and downstream of the TSS of each gene. The annotated peaks were classified as “K4 only,” “K27 only,” “bivalent” (including both overlapping H3K4me3 and H3K27me3 marks), and other classes. We identified 36.1%, 32.3%, and 24.4% of genes associated with “K4 only” in NSCs, OPCs, and NFOs, respectively, and 3.5%, 5.4%, and 3.8% of genes associated with “K27 only” in NSCs, OPCs. and NFOs, respectively. In contrast, only 4.7%, 2.8%, and 1.5% of genes contained bivalent domains in NSCs, OPCs, and NFOs, respectively (Fig. 4c, Additional file 5: Table S4). Histone methylation states were highly correlated to gene expression (Fig. 4d–f), as genes containing only H3K4me3 or H3K27me3 marks showed higher and lower expression levels, respectively, in all cell types, and genes containing bivalent domains showed intermediate expression. Additionally, we also examined some other oligodendrocyte-related genes using the histone ChIP-Seq and RNA-Seq results (e.g., *Nkx2-2*, *Olig2*, *Yy1*, and *Gpr17*) (Fig. 4g and Additional file 6: Fig S2). For example, *Olig2* plays important role in the initial specification of oligodendrocyte lineage [12, 50], and *Nkx2-2*, *Yy1*, and *Gpr17* are subsequently required for the generation of mature oligodendrocytes [51–53].

### The dynamic regulation of bivalent genes during oligodendrocyte formation and differentiation

**Fig. 5:**
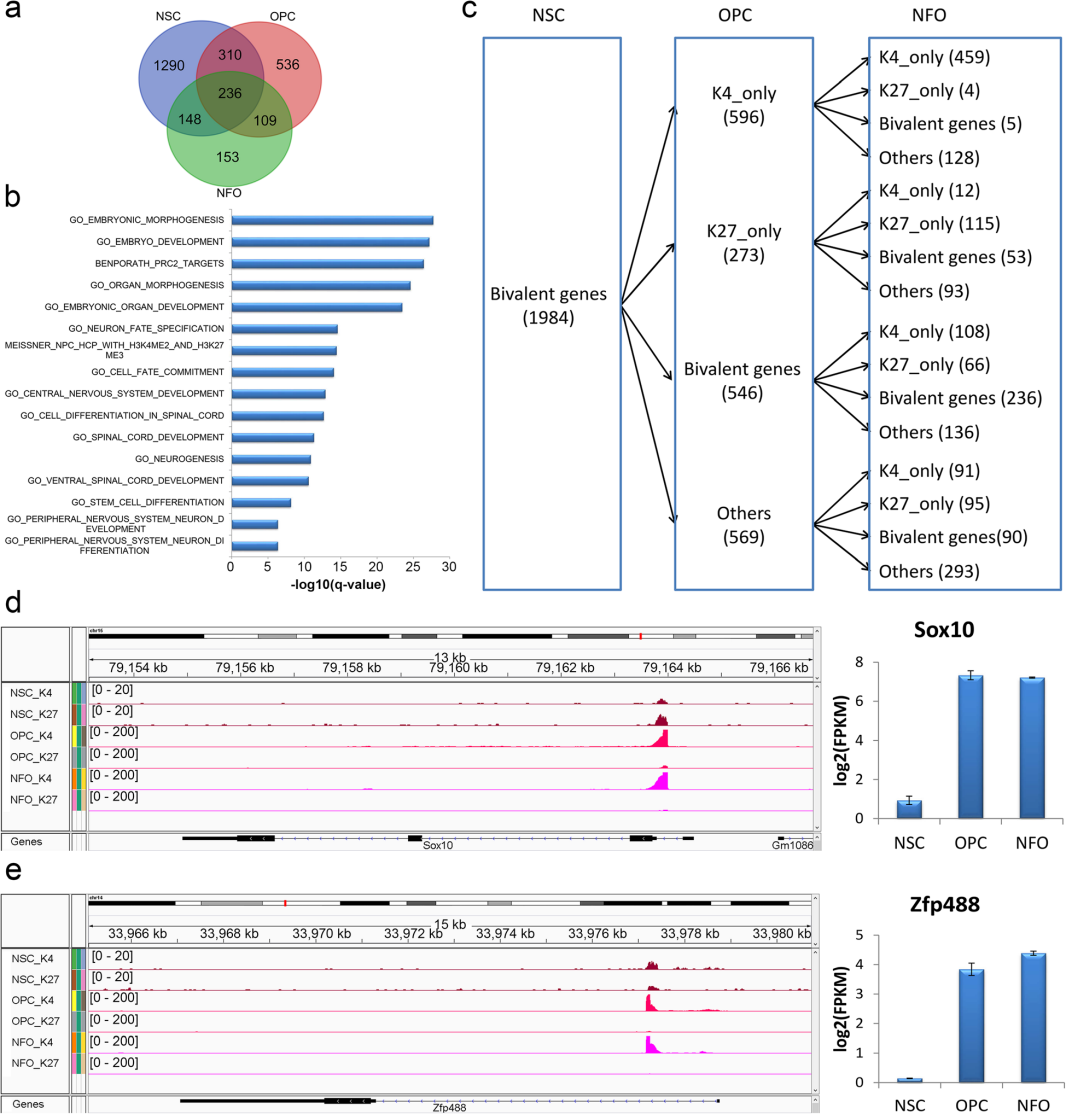
Bivalent genes in NSCs, OPCs, and NFOs. **a** Overlaps of bivalent genes in NSCs, OPCs, and NFOs. **b** Functional enrichment of overlapping bivalent genes in NSCs, OPCs, and NFOs, based on MSigDB. **c** Dynamic regulation of NSCs bivalent genes in OPCs and NFOs. **d**, **e** H3K4me3 and H3K27me3 binding peaks and gene expression of *Sox10* (**d**) and *Zfp488* (**e**)

Bivalent domains were initially found to keep genes silent or expressed at very low levels yet poised for activation in ESCs [54, 55]. The number of bivalent genes (including both coding and non-coding genes) decreased during differentiation of oligodendrocytes (1984 genes in NSCs, 1191 genes in OPCs and 646 genes in NFOs) (Figs. 4c and 5a). Comparing NSCs, OPCs, and NFOs, we found 236 common bivalent genes in all cell types (Fig. 5a). These common bivalent genes showed strong functional enrichment in “embryonic morphogenesis,” “central and peripheral nervous system development,” “stem cell differentiation,” and “neurogenesis” (Fig. 5b).

Next, we investigated the dynamics of regulation of bivalent genes during the transition of NSCs to OPCs and NFOs (Fig. 5c). A total of 1984 bivalent genes were analyzed in NSCs. Among these, 23.1% (459) had “K4 only” marks in OPCs and NFOs. For example, we found that *Sox10* and *Zfp488* were bivalent genes in NSCs and had “K4 only” marks in OPCs and NFOs (Fig. 5d, e). The expression levels of these genes were low in NSCs but high in OPCs and NFOs (Fig. 5d, e). It has been found that *Sox10* may induce NSCs to enter the oligodendrocyte lineage [56] and *Zfp488* may promote oligodendrocyte differentiation in adult subventricular zone (SVZ) neural stem/progenitor cells (NSPCs) [57].

### OLIG2 is involved in regulation of its own expression at the transcriptional level and after transcription

**Fig. 6:**
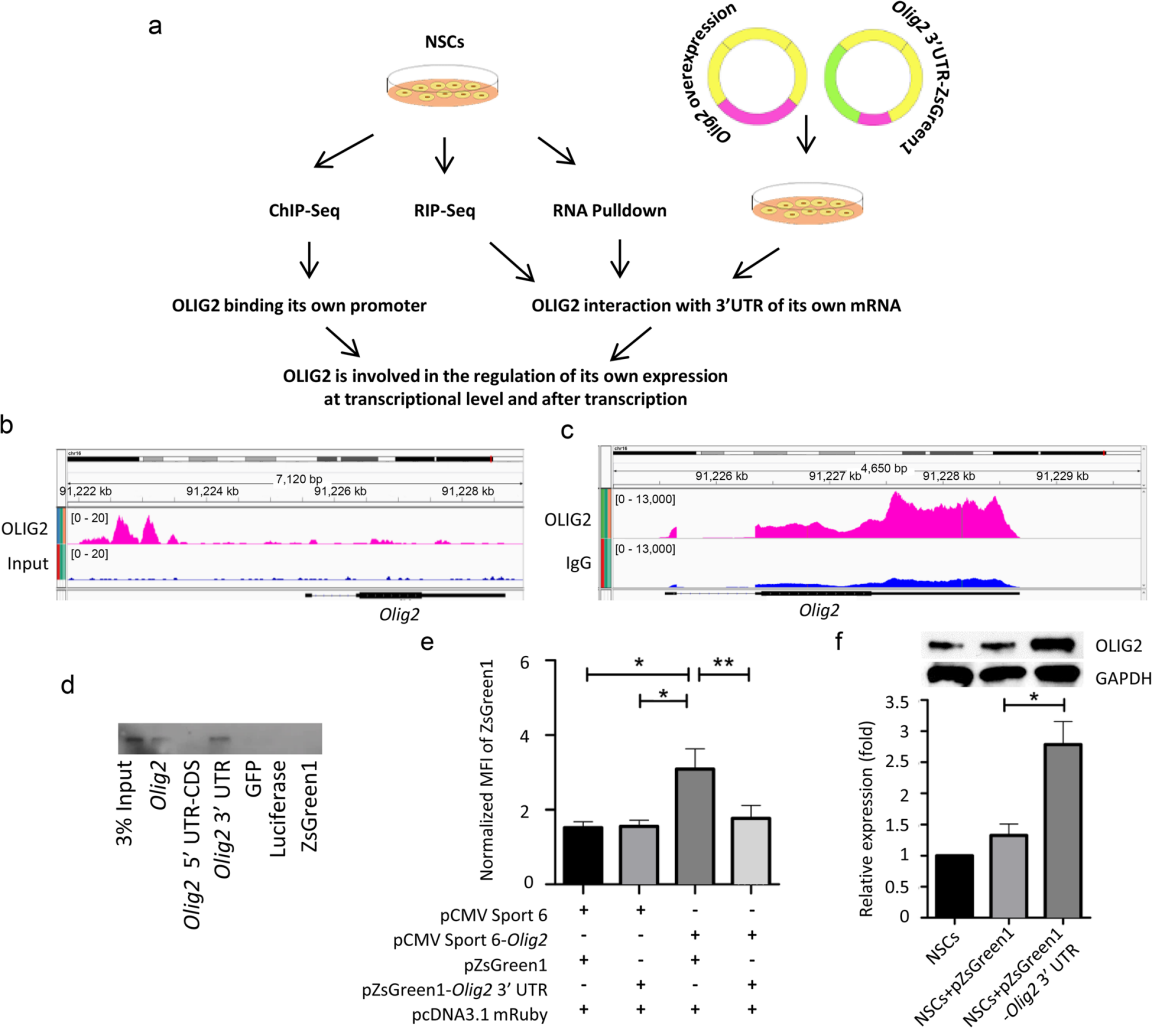
OLIG2 is involved in regulation of its own expression at the transcriptional level and after transcription. **a** Experimental overview. **b** ChIP-Seq result shows that OLIG2 binding to its promoter region, potentially self-regulates its own transcription. **c** Binding of OLIG2 to *Olig2* mRNA in NSCs shown by RIP-Seq. IgG was used as control. **d** In vitro transcribed biotinylated *Olig2* RNA retrieves OLIG2 protein. The profiles were established by RNA protein pull-down using NSCs protein extract. Retrieved OLIG2 protein (32 kDa) was detected by Western blot assay. **e** Addition of the *Olig2* 3′ UTR to the ZsGreen1 reporter C1 end substantially reduces expression of ZsGreen1 in *Olig2* overexpressing cells. Flow cytometric analyses on MFI of ZsGreen1 fluorescence signal in 293FT cells 48 h after transfection. The MFI of ZsGreen1 fluorescence signal was normalized by dividing the MFI of mRuby fluorescence signal in each replicate. The error bars show standard errors of the means (error bars) (*n* = 6 in each assay) **f**. The normalized protein level of OLIG2 in NSC cells 48 h after transfected ZsGreen1 reporter constructs with or without *Olig2* 3′ UTR. The protein level of OLIG2 was examined by Western blot and normalized to corresponding GAPDH levels. All quantified data were presented as mean ± SEM (standard error of the mean). *N* = 3; *t* test analysis *, *p* < 0.05; **, *p* < 0.01

To investigate the self-regulation of OLIG2, we performed a series of experiments as shown (Fig. 6a). Our ChIP-Seq results show that OLIG2 could bind the promoter of *Olig2* (Fig. 6b) in NSCs, suggesting that OLIG2 potentially self-regulates its own transcription; however, whether OLIG2 can be involved in regulation after transcription has not been previously demonstrated. Thus, we performed a RIP-Seq (RNA immunoprecipitation-sequencing) assay in NSCs (Fig. 6c). As shown in Additional file 7: Table S5, we found 11 potential binding sites with significant called peaks in OLIG2. Importantly, *Olig2* 3′ UTR is the most significant target with a tag fold-change of 18 (OLIG2 vs IgG) and *p*-value = 1.65e−09. To further verify the binding of OLIG2 to its own mRNA, we performed an RNA protein pull-down assay (Fig. 6d). Equal amounts of *in vitro* transcribed biotinylated RNAs including full-length of *Olig2*, 5′ UTR and coding sequence only (CDS) of *Olig2*, *Olig2* 3′ UTR*,* GFP, ZsGreenC1, and luciferase (negative controls) were incubated with NSCs protein extracts, respectively. RNAs were purified with streptavidin-coated beads and bound proteins were analyzed by Western blot. We found that only purified full-length *Olig2* RNA and *Olig2* 3′ UTR with biotin labeling could retrieve OLIG2 protein, but 5′ UTR-CDS of *Olig2*, GFP, ZsGreenC1, and luciferase RNA could not (Fig. 6d).

Given that *Olig2* likely associated with its own mRNA, we sought to examine the effect that overexpressing *Olig2* could have on the expression of a ZsGreen1 reporter under the control of the 3′ UTR of *Olig2*. To do this, ZsGreen1 reporter constructs with or without *Olig2* 3′ UTR were created (Additional file 8: Fig S3). The pcDNA3.1 mRuby plasmid was used as an internal control for the normalization of transfection efficiency. In addition to the pcDNA3.1 mRuby vector, ZsGreen1 reporter constructs were co-transfected with pCMV-Sport 6-*Olig2* vector or empty vector pCMV-Sport 6 into 293FT cells at equal molar ratios respectively. Median Fluorescent Intensity (MFI) of ZsGreen1 and mRuby were measured by flow cytometry. Considering transfection efficiency variation, the MFI of ZsGreen1 signal was normalized by dividing the MFI of mRuby in each replicate (n = 6) (Fig. 6e). Flow cytometry results indicated the normalized MFI from ZsGreen1 was similar to that from ZsGreen1-*Olig2* 3′ UTR when co-transfected with the empty control vector pCMV-Sport 6. However, the normalized MFI from ZsGreen1 was upregulated in cells with co-transfection of the pCMV-Sport 6-*Olig2* vector, possibly due to the activation effect of OLIG2 overexpression. The expression of ZsGreen1 construct could be regulated by OLIG2 or by other molecules regulated by OLIG2 in 293FT cells. Importantly, the inclusion of *Olig2* 3′ UTR in the ZsGreen1 reporter significantly inhibited the normalized MFI from ZsGreen1 induced by OLIG2 overexpression, making the normalized MFI comparable to that in cells without OLIG2 overexpression. This indicated that the addition of the *Olig2* 3′ UTR to the C1 end of the ZsGreen1 reporter construct led to a significant reduction in ZsGreen1 protein in the *Olig2*-overexpressed cells compared with the ZsGreen1 construct without the 3′ UTR of *Olig2* (Fig. 6f); thus, OLIG2 protein inhibited ZsGreen1 reporter gene expression controlled by the *Olig2* 3′ UTR.

Furthermore, as the hypothesis is that OLIG2 protein can inhibit its expression by binding to the *Olig2* 3′ UTR, we examined if overexpressing *Olig2* 3′ UTR can influence OLIG2 expression, leading to the changes in the endogenous protein level of OLIG2 in NSCs. As such, we performed nucleofection of the NSCs with ZsGreen1 reporter constructs with or without *Olig2* 3′ UTR. Two days after nucleofection, cells were harvested for Western blot analysis for the endogenous protein levels of OLIG2 and GAPDH. GAPDH acted as an internal control and was used to normalize the amount of protein lysate loaded for Western blot. The signals from Western blot were quantified with ImageJ for Western blot densitometry analysis. The densitometry values of OLIG2 were normalized by dividing that of GAPDH. As shown in Fig. 6g, the normalized protein level of OLIG2 was significantly higher in cells transfected with the ZsGreen1-*Olig2* 3′ UTR vector than that transfected with ZsGreen1 vector, suggesting the expression of endogenous OLIG2 protein can be influenced by *Olig2* 3′ UTR, possibly due to its competitively inhibitory effect on the endogenous *Olig2* mRNA for OLIG2 protein interaction.

Taken together, these results suggest that OLIG2 self-regulates at the transcriptional step (Fig. 6b) and after transcription through the 3′ UTR of its own mRNA (Fig. 6d–f).

## Discussion

Oligodendrocyte development is a complex biological process encompassing multiple stages. Previous studies have indicated that *Olig2* plays essential roles in neural progenitor proliferation and differentiation through direct regulation of gene expression or cooperation with other TFs and cofactors [58–61]. Many of the protein coding target genes that function in oligodendrocyte differentiation have been reported but few of the involved lncRNAs are known. In our study, we found that lncRNAs regulated by *Olig2* are functionally enriched during oligodendrocyte differentiation. Furthermore, bivalent genes are evidently important in pluripotent stem cell self-renewal and differentiation. Herein, we have characterized the dynamics of bivalent genes during oligodendrocyte differentiation and shown that some bivalent genes may be regulated by *Olig2*. Although many previous studies of *Olig2* focused on the process of transcription, little is known about its role in regulation after transcription. Interestingly, our experiments showed that OLIG2 not only regulates its own transcription, but is also involved in regulation after transcription via the 3′ UTR of its own mRNA.

lncRNAs play important functional roles during oligodendrocyte formation and differentiation, as our study and others have previously demonstrated [21, 22]. The pioneering studies of Mercer et al. used custom-designed microarrays to show that a number of known lncRNAs exhibit differential expression during oligodendrocyte lineage specification, providing early evidence for lncRNAs in NSCs fate decisions and oligodendrocyte lineage maturation [62]. However, incomplete annotation of lncRNAs at the time hindered interrogation of novel lncRNAs and no experiments were performed to demonstrate the functional significance and mechanisms of lncRNAs beyond changes in their expression [63, 64]. Our preliminary motif analyses with DNase-DGF (Digital DNase Genomic Footprinting) data and Mouse ENCODE (The Encyclopedia of DNA Elements) promoters showed that many other OPC-enriched lncRNAs may be regulated by OLIG2 [21, 34, 43]. Importantly, we have demonstrated that gene knockdown of our top candidate, *lnc-OPC*, significantly reduces OPCs differentiation from NSCs. OLIG2-binding sites in the upstream regulatory region of *lnc-OPC* have been identified in ChIP-Seq experiments and validated using luciferase assays. Notably, after our work focusing on OPCs formation was published in PLOS GENETICS, He et al. also published their interesting study demonstrating the important functional role of lncRNAs during oligodendrocyte differentiation stages, and cited the utilization of our databases for purified cells and spinal cord injury [22]. Thus, lncRNAs might be central to a new paradigm that defines oligodendrocyte development.

Our previous study showed that OLIG2 could bind and repress *lnc-OPC* expression in NSCs, likely to maintain NSC self-renewal [21]. In the current study, we found that OLIG2 did not bind to the upstream regulatory region of *lnc-OPC* in OPCs. Thus, *lnc-OPC* was no longer inhibited by OLIG2 and *lnc-OPC* expression level significantly increased in OPCs [21]. As we described, knocking down *lnc-OPC* greatly reduced the number of OPCs formed from NSCs suggesting that *lnc-OPC* is critical for OPCs formation. Besides *lnc-OPC*, in this study, we detected a total of 613 expressed lncRNAs that have OLIG2 called peaks in NSCs, OPCs, or NFOs, and that clustered according to their distinct expression patterns, as shown in Fig. 3a. Genes from different clusters might play different roles in oligodendrocyte formation and differentiation. For instance, *Crnde* is grouped into cluster 5 and bear H3K4me3 marks in NSCs. According to the gene expression patterns of lncRNAs in eight purified brain cell types that we have published, *Crnde* appears to be an NSC-specific gene (Additional file 9: Fig S4a). This observation is consistent with previous reports of the role of *Crnde* in the maintenance of cell pluripotency and its high expression in iPSCs (inducible pluripotency cells) [62, 65]. Moreover, we found *Crnde* with OLIG2 called peaks in OPCs and NFOs, but not in NSCs, suggesting that *Crnde* may be repressed by *Olig2* in the oligodendrocyte lineage. On the other hand, in cluster 7 we found lncRNAs with lower expression in NSCs than in OPCs and NFOs, suggesting these lncRNAs might play roles in oligodendrocytes differentiation (Fig. 3a). Combining the results of our gene expression analyses with the observed OLIG2-binding patterns in NSCs, OPCs, and NFOs indicates that OLIG2 may act as a repressor of 39 lncRNAs in NSCs (group 1) and act as an activator of nine lncRNAs in both OPCs and NFOs (group 2). Our functional enrichment analysis showed that the expression of group 1 and 2 genes were positively correlated with oligodendrocyte development and differentiation (Fig. 3b). The above results suggest that *Olig2* potentially regulates oligodendrocyte differentiation through activation or repression of lncRNA expression. It is our future goal to test the results and hypotheses from these correlative analyses.

In an independent study, *Olig2* was overexpressed in neural progenitor cells. We re-analyzed RNA-Seq data from Nishi et al. and identified genes that were differentially expressed after *Olig2* overexpression [66]; some genes have OLIG2 binding motifs in our studies. For example, expression of *Gm28513* was up-regulated upon overexpression of *Olig2* (log2 (fold-change) > 2.4; *p* value < 0.001). Interestingly, our ChIP-Seq data indicates that *Gm28513* has OLIG2 called peaks only in NSCs but not in oligodendrocyte lineages (Additional file 9: Fig S4b) and has significantly higher expression in NSCs compared with OPCs (log2(fold-change) > 4.8; *p* value < 0.001). The function of lncRNAs predicted by “guilt by association” indicated that *Gm28513* was enriched in the MSigDB functional term “oligodendrocyte differentiation” (NES = − 1.41). Collectively, these results suggest that *Gm28513* may be activated by *Olig2* and might function in NSCs self-renewal.

OLIG2 is a master regulator of oligodendrocyte fate determination that is essential for NSCs self-renewal and oligodendrocyte specification from NSCs, and that exerts diverse functions at different stages. Additionally, the differentiation of OPCs into mature oligodendrocytes is also regulated by OLIG2 [67]. In the current study, we identified putative *Olig2*-regulated genes at different stages of oligodendrocyte differentiation based on our ChIP-Seq and RNA-Seq datasets across NSCs, OPCs, and NFOs. In our studies, we inferred potential OLIG2 activated/repressed genes based on OLIG2 called peaks and gene expression. H3K4me3 and H3K27me3 are only two of the chromatin marks and there are other activated/repressed chromatin marks. For example, to determine which OLIG2 called peaks are associated with an active enhancer mark, such as H3K27 acetylation, and test whether called peaks involved in repression may tend to lack this mark, we searched the literature for H3K27ac data. Bertolini et al. [68] and Wang et al. [69] identified OLIG2-activated genes in NSCs based on H3K27ac marks. We re-analyzed these data and compared the potential OLIG2 activated/repressed genes based on OLIG2 called peaks and gene expression with the H3K27ac peak positions. The results showed among these datasets, 51.8–62.1% of the inferred OLIG2-activated peaks were overlapping with H3K27ac called peaks. Thereby, potentially activated genes/peaks were more frequently associated with active enhancer mark such as H3K27ac, than potentially repressed genes/peaks by OLIG2.

During the transition from NSCs to OPCs, we found that the numbers of upregulated and downregulated genes annotated with OLIG2 called peaks were similar in each cell type (Fig. 2a, c). However, during the transition from OPCs to NFOs, more OLIG2 called peaks belong to downregulated than upregulated genes (Fig. 2b, d). NFOs are newly formed oligodendrocytes, the intermediate stage in the transition from OPCs to mature oligodendrocytes [43]. Our data showed that upregulated genes in NFOs annotated with OLIG2 called peaks were enriched in “oligodendrocyte differentiation” and that downregulated genes in NFOs were enriched in “neuron” and “neurogenesis” (Fig. 2f). Therefore, *Olig2* might both promote oligodendrocyte differentiation and repress neuronal fate in OPCs. One potential caveat in our ChIP-Seq analysis is that we only report called peaks instead of differential binding at different stages. Due to technical limitations, loci without peaks in a sample do not imply the absence of regulation at a particular stage.

During oligodendrocyte lineage specification, a number of stage-specific TFs control oligodendrocyte specification, differentiation, and myelination [8]. We characterized genome-wide OLIG2 occupancy across different stages of oligodendrocyte differentiation and its potential cooperation with other TFs to regulate oligodendrocyte development. Consistently, previous studies have shown that OLIG2 could function together with ALCL1 and further promote the differentiation of oligodendrocyte progenitors into oligodendrocytes [70]. Moreover, our data suggest that other TFs (MYC and SMAD2/3) occupy the overlapping OLIG2 binding regions. MYC motifs are enriched in the down-regulated genes in OPCs vs. NSCs and NFOs vs. OPCs, which is consistent with the reports that MYC could repress oligodendrocyte differentiation [71, 72], and that the loss of *Smad3* at E12.5 led to a severe oligodendrocyte phenotype including decreases in the numbers of progenitors and mature cells, and delay of myelination [73]. SOX family proteins are important transcription regulators [74, 75]. We found SOX2 and SOX10 motifs were enriched in the OLIG2 called regions. Due to the relatively limited ability of motif-finding programs to discriminate SOX family of proteins and their overlapping functions, SOX proteins such as SOX8 and SOX9 may also potentially cooperate with OLIG2 in regulating oligodendrocyte development.

The interactions of TFs with chromatin and modified histones determine gene expression. Our genome-wide H3K4me3/H3K27me3 ChIP-Seq analysis in NSCs, OPCs, and NFOs showed that H3K4me3 or H3K27me3 marks were gained or lost in a specific group of genes at different stages of oligodendrocyte differentiation, which implies that these genes could dynamically be regulated and may function at specific stages of oligodendrocyte differentiation. Bivalent domains occupied by both H3K4me3 and H3K27me3 marks have important roles in pluripotency and cell differentiation. For example, genes with bivalent domains in their promoters are silent yet poised to be activated during the differentiation of ESCs [40]. However, little is known about the function of bivalent genes during oligodendrocyte development. We found that bivalent genes were dynamically regulated during the process of oligodendrocyte differentiation (Fig. 5a). In particular, we found that the promoter regions of 459 genes that had bivalent domains in NSCs then presented “K4 only” marks in the oligodendrocyte lineage cells OPCs and NFOs. The gene expression patterns of 367 of these 459 genes detected at least in one cell type (FPKM > 1) were then analyzed by the k-means clustering method (Additional file 10: Fig S5a). Six Clusters were generated and the expression of 94 genes in cluster 2 had increased in OPCs and NFOs (Additional file 10: Fig S5a), which suggests that these genes might be activated during the differentiation of NSCs into oligodendrocyte lineage cells. Additionally, our results also showed that promoter regions of 108 genes contained bivalent domains in NSCs and OPCs but exhibited “K4 only” marks in NFOs (Additional file 10: Fig S5b). The gene expression patterns of 72 of these 108 genes were analyzed using the same method with the former. Six Clusters were generated and three genes (*Epcam*, *Pdk1*, and *Cbln2*) in cluster 4 were expressed at lower levels in NSCs and OPCs than in NFOs (Additional file 10: Fig S5b). The results suggested that these genes might become activated after the OPCs stage and thereby promote OPC differentiation into oligodendrocytes.

The bivalent genes in NSCs began to be activated at different stages (OPCs or NFOs) after NSCs. To investigate whether the activated genes might be regulated by OLIG2, we correlate the OLIG2 ChIP-Seq with RNA-Seq in NSC, OPC, and NFO. Our data showed that 13 bivalent genes of the 94 activated genes (*Pcdh15*, *Ptprk*, *Tmem132d*, *Mkrn1*, *Fbll1*, *Slc1a1*, *Kif26a*, *Ugt8a*, *2210016F16Rik*, *Bnip3l*, *Rell1*, *Taok3*, and *Cadm2*) had OLIG2 called peaks in OPCs and NFOs, but not in NSCs. These genes were also highly expressed in OPCs and NFOs, which suggests that they might be activated by OLIG2 in OPCs and NFOs and subsequently function in oligodendrocyte differentiation. In particular, the activated gene *Epcam* had OLIG2 called peaks only in NFOs, which suggests that it might be regulated by OLIG2 and function during OPCs differentiation into NFOs. Together, these results suggest that the dynamic modification of bivalent genes could have essential roles in oligodendrocyte differentiation and development and that some genes might be regulated by OLIG2 during this process. A potential caveat of our bivalent genes’ analysis is that the overlap of H3K27 and H3K4 methylation is not at the single cell level. As there could be multiple subpopulations within our isolated cells, single cell experiments are needed in the future to comprehensively study the dynamics of gene activation and repression.

One of the potential mechanisms by which OLIG2 could regulate oligodendrocyte development is through lncRNAs. We know that lncRNAs often bind to chromatin modification complexes to guide them to specific locations in the genome, changing epigenetic landscapes and controlling downstream genes to define cell fate. Our experiments showed that *lnc-OPC* was bound to EZH2 and SUZ12 in NSCs (Additional file 11: Fig S6). EZH2 and SUZ12 are two subunits of the PRC2 chromatin modification complex [76], and He et al. have also demonstrated that *lncOL1* and SUZ12/PRC2 could form a complex to increase expression of oligodendrocyte differentiation-related genes to promote oligodendrocyte differentiation [22].

The basic helix-loop-helix (bHLH) proteins have been reported to act as RNA-binding protein previously [77]. For example, the helix-loop-helix (HLH) domain has been identified as an RNA-binding motif in eukaryotic translation initiation factor 3 (eIF3) [77]. eIF3 can not only regulate translation of hepatitis C virus RNAs by binding to the Internal Ribosome Entry Site (IRES) but also control translation of oncogenic MYC gene by interacting with its 5′-UTR elements [77]. Our OLIG2 RIP-Seq data in NSCs showed that the most significant OLIG2 target was its own 3′ UTR. An RNA protein pull-down assay was performed and confirmed that OLIG2 could interact with *Olig2* mRNA. This is consistent with previous reports in the literature showing that DNA-binding TFs can also interact with RNAs in some cases [78]. For example, Yin-Yang 1 (YY1), a well-known TF in the regulation of oligodendrocyte progenitor differentiation, can bind to both DNA regulatory elements and RNAs of its target genes [52]. The tethered RNA species reinforce YY1 occupancy at these elements and stabilize the gene expression process [52]. Intriguingly, some proteins have autoregulation potential: they can function in a feedback loop by binding to the ARE in their own mRNA to regulate mRNA processing, stability, or translational efficiency [52, 78–80]. For instance, TDP-43 and the HTT protein interact with several ARE in their own mRNA to regulate their own expression [41, 42]. In our study, we also found several AREs in the 3′ UTR of the *Olig2* mRNA (Additional file 12: Fig S7a) using AREsite, an online database for the identification of AU-rich elements [81], and evolutionary conservation analysis indicated that these AU-rich regions are highly conserved across multiple species including mouse and human (Additional file 12: Fig S7b). Additionally, we observed the expression of endogenous OLIG2 protein can be influenced by *Olig2* 3′ UTR. These results suggest *Olig2* 3′ UTR may directly or indirectly interact with OLIG2 protein by competing endogenous *Olig2* mRNA. Whether this interaction is direct or indirect still needs further investigation. Also, we cannot rule out the possibility that 3′ UTR can sequester other molecules that may reduce OLIG2 expression such as microRNAs and this can be tested in the future.

Through the analysis of OLIG2 RIP-Seq data, we found that OLIG2 significantly binds other targets (e.g. *Noc2l*, *Rae-1*, and *Rfc3*) with reported relevant functions in NSC biology. Using single cell transcriptomics researchers found *Noc2l*, an inhibitor of histone acetyltransferases, to be an early neural progenitor marker [82]. In another study *Rae-1*, the retinoic acid early induced transcript was found to be involved in regulating adult SVZ by supporting progenitor cell proliferation [83]. Interestingly *Rfc3*, the replication factor C subunit 3 is a predicted target gene of OLIG2 in the CHEA Transcription Factor Targets dataset from the Harmonizome database [84]. These results suggest that OLIG2 contributes to NSC proliferation by regulation after transcription. Further experiments are required to validate the binding of OLIG2 to other target mRNAs and the functional implications of these regulatory mechanisms in NSCs.

## Conclusions

**Fig. 7:**
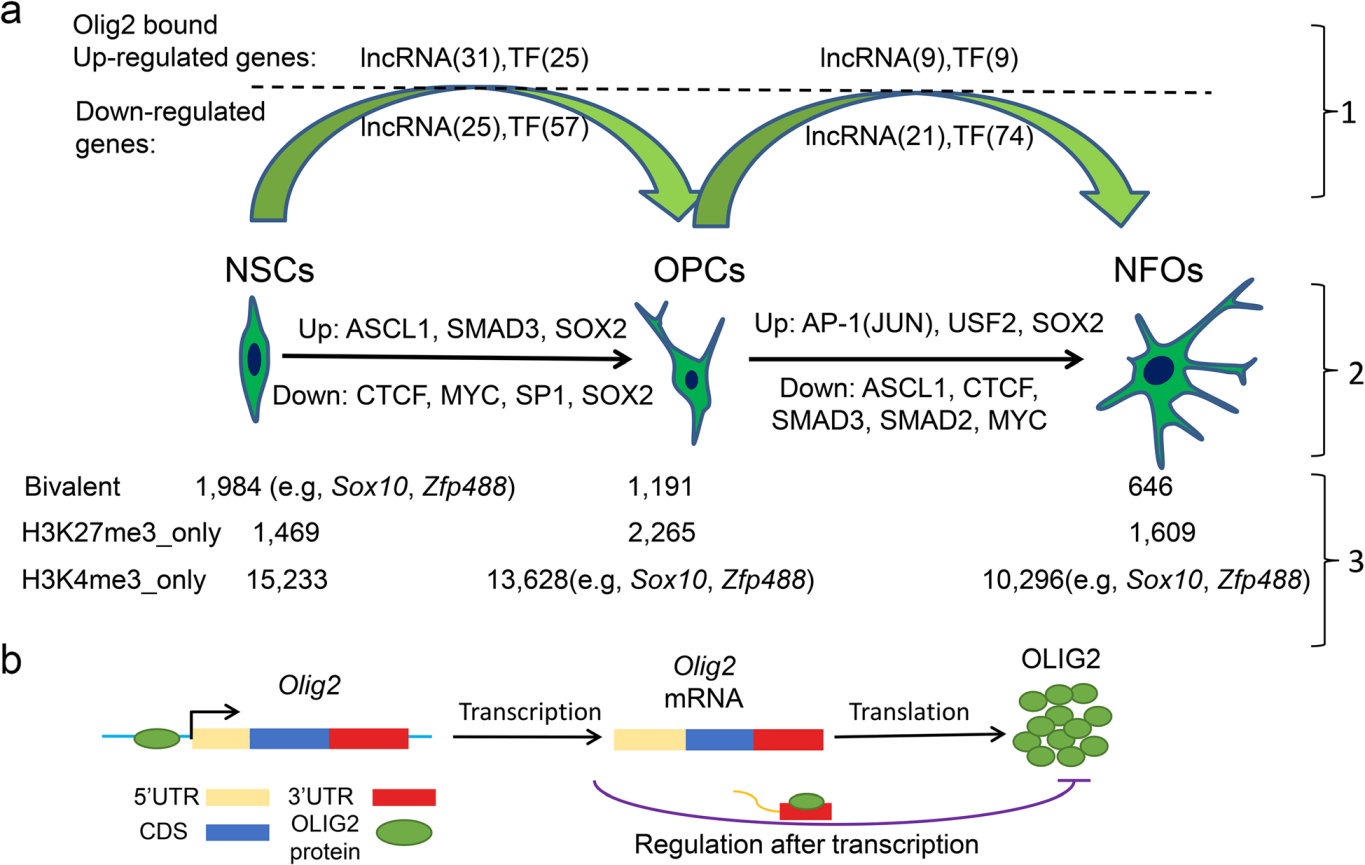
Diagram summarizing the main findings. **a** The first layer indicates the up- and downregulated of lncRNAs and TFs with OLIG2 called peaks. The second layer shows some examples of the TFs potentially cooperating with OLIG2 in the regulation of gene expression. The third layer indicates the dynamically regulated genes with histone marks in NSCs, OPCs, and NFOs and some examples mentioned in the manuscript. **b** The proposed involvement of OLIG2 in regulating its own gene expression at the transcriptional level as a transcriptional factor, and after transcription through *Olig2* 3′ UTR directly or indirectly to influence OLIG2 expression

Collectively, our genome-wide study of OLIG2 binding and epigenetic modification status during multiple stages of oligodendrocyte differentiation enables insights into stage-specific regulation of oligodendrocyte differentiation and reveals the previously unknown auto-regulation of OLIG2 expression at the transcriptional level and after transcription (Fig. 7). In the future, it would be essential to study the downstream genes and pathways regulated by these lncRNAs and to construct a genome-wide TF-lncRNA-chromatin modification protein regulatory network controlling oligodendrocyte development. These studies reveal potential missing links in the mechanisms of oligodendrocyte development, thereby can advance our understanding of the fundamental cell fate decisions during NSCs differentiation, demyelination diseases, and regenerative medicine.

## Methods

### Cell culture

The investigators conducting the experiments were blinded to group assignment and outcome assessment for all the following experiments described in this article.

Mouse neural stem cells (NSCs) from the cortices of embryonic (E14.5) CD-1 mice were purchased from R&D Systems. Briefly, cells were cultured on poly-d-lysine-coated flasks and passaged every 2 days in MEM/F12 medium (Gibco) containing 20 ng/ml EGF, 20 ng/ml FGF, B27, and N2 supplement (Invitrogen). When passaging, cells were dissociated using Accutase (Invitrogen). 293FT cell line was purchased from ATCC. Cells were cultured in DMEM (Gibco) medium with 10% FBS (Fisher) and passaged every 2–3 days.

### RNA immunoprecipitation and RNA sequencing

RNA-binding protein immunoprecipitation was carried out using an EZ-Magna Nuclear RIP (Cross-Linked) Nuclear RNA-Binding Protein Immunoprecipitation Kit (Catalog No. 17-10521, Millipore). Briefly, NSCs were fixed in 0.3% formaldehyde for 10 min at room temperature and quenched with glycine. Cells were collected by scraping and then pelleted by centrifugation. Cells were lysed in Nuclei Isolation Buffer and sonicated using a Bioruptor device (Diagenode) with a program of 5 cycles of 30 seconds ON and 30 seconds OFF. Sheared cross-linked chromatin was treated with DNaseI and immunoprecipitated using anti-OLIG2 antibody (AB9610, EMD Millipore) or rabbit IgG (2729S, Cell Signaling Technology) coupled to Magna ChIP protein A/G Magnetic Beads with rotation overnight at 4 °C. Protein-RNA cross-links were reversed by incubating at 60 °C in Elution Buffer. RNA was extracted using TRIzol (15596026, Invitrogen) and was used for library construction with the TruSeq RNA Library Prep Kit (RS-122-2001, Illumina) following the manufacturer’s protocols.

RIP to analyze the interactions between *lnc-OPC* and EZH2/SUZ was performed using EZ-Magna Nuclear RIP (Cross-Linked) Nuclear RNA-Binding Protein Immunoprecipitation Kit (Catalog No. 17-10521, Millipore) with NSCs lysate and anti-EZH2 or anti-SUZ12 as the immunoprecipitating antibody. IgG immunoprecipitation was used as a negative control. EZH2 antibody was purchased from EMD Millipore (17-662). SUZ12 antibody was purchased from Abcam (ab12073). Mouse IgG antibody was purchased from EMD Millipore (12-371).

### Animal experiments

Postnatal day 7 mouse brain (C57BL/6; The Jackson Laboratory, Bar Harbor, ME) were used for OPCs and NFOs purification. All animal experiments were performed according to the University of Texas Health Science Center Animal Welfare Committee guidelines for laboratory animals. We utilized sex-matched (approximately half male and half female) mice in all studies.

### Purification of oligodendrocyte progenitor cells (OPCs) and newly formed oligodendrocytes (NFOs)

OPCs and NFOs were acutely purified from mouse brains by immunopanning as described previously [34, 43, 44]. Briefly, mouse brains were dissociated into single cell suspensions with Neural Tissue Dissociation Kit (P) (MACS Miltenyi Biotec; 130-092-628) according to detailed manufacturer’s instructions. After depletion of microglia and endothelial cells from dissociated single cell suspensions with BSL-1 coated plates, the remaining cells were incubated on rat-PDGFRα antibody-coated (BD Pharmingen; 558774) plates to harvest OPCs and then incubated on a plate coated with anti-GalC hybridoma supernatant (from Dr. Qinlin Cao laboratory) to harvest NFOs.

### ChIP and sequencing

OLIG2 Low-cell ChIP was performed using the True MicroChIP Kit (Diagenode) and libraries were constructed with the DNA SMART ChIP-Seq Kit (Clontech) by following the manufacturer’s instructions. OLIG2 antibody used for ChIP-Seq was purchased from EMD Millipore (AB9610). Rabbit IgG unconjugated polyclonal antibody used for ChIP-Seq was purchased from Cell Signaling Technology (2729S). EZH2 antibody was purchased from EMD Millipore (17-662). SUZ12 antibody was purchased from Abcam (ab12073). Mouse IgG antibody was purchased from EMD Millipore (12-371).

### RNA pull-down assay

RNA pull-down assay was performed as previously described [85]. The full-length *Olig2* DNA was prepared by linearization of *Olig2* plasmid DNA by PacI enzyme, and *Olig2* 5′ UTR-CDS was linearization of *Olig2* plasmid by AfeI enzyme. The *Olig2* 3′ UTR and ZsGreen1 sequences were obtained by PCR amplification. The pRS314-T7-GFP and T7-CMVtrans-FFLuc-polyA plasmids were linearized by restrictive enzyme Kpnl and SacI respectively. Biotin-labeled RNAs were transcribed in vitro using the Biotin RNA Labeling Mix (Roche) and T7 RNA polymerase (Promega). The biotinylated RNAs were treated with RNase-free DNase I (Promega) and purified using the RNeasy Minielute Cleanup Kit (QIAGEN). Three micrograms of RNA were heated to 90 °C for 2 min, put on ice for 2 min in RNA Structure Buffer (10 mM Tris pH 7, 0.1 M KCl, 10 mM MgCl2), and then incubated at room temperature (RT) for 20 min to allow proper secondary structure formation. 10^7^ NSCs were harvested from culture by centrifugation at 300 g for 5 min. The cell pellet was resuspended in 2 ml PBS, 2 ml Nuclear Isolation Buffer (1.28 M sucrose, 40 mM Tris-HCl pH 7.5, 20 mM MgCl2, 4% Triton X-100), and 6 ml sterile water) on ice for 20 min. Nuclei were isolated by centrifugation at 2500*g* for 15 min. The nuclear pellet was resuspended in 1 ml RIP buffer (150 mM KCl, 25 mM Tris pH 7.4, 0.5 mM DTT, 0.5% NP40, 1 mM PMSF, and protease inhibitor (Roche Complete Protease Inhibitor Cocktail Tablets)). A Dounce homogenizer was used to mechanically shear the nuclei with 15–20 strokes followed by centrifugation at 13,000 RPM for 10 min to remove nuclear debris. Approximately 3% of the supernatant was reserved as the input sample. Structured RNA was then mixed with the NSCs nuclear extract in RIP buffer and incubated at RT for 1 h. A 60-μl aliquot of Streptavidin Agarose Beads (Invitrogen) was added to each reaction, which was further incubated at RT for 1 h. Beads were washed five times using Handee spin columns (Pierce) and boiled in SDS protein loading buffer, and standard Western blot technique [86] was used to detect OLIG2 protein. Scramble RNA was used as a negative control.

### Antibodies and plasmids

Rabbit anti-OLIG2 antibody was purchased from Millipore, CA (Cat. AB9610). Anti-GAPDH antibody was purchased from Millipore (Cat. ABS16). Pierce Fast Western Kit was purchased from Thermo Fisher Scientific Inc, MA (Cat. 35601). The PCR-cloned full-length *Olig2* 3′ UTR sequence from NSCs cDNA was sub-cloned downstream of pZsGreen1-C1 (Clontech) plasmid to generate the ZsGreen1 reporter constructs. The pCMV-Sport6 plasmid was purchased from Horizon Discovery and the *Olig2* cDNA was purchased from Dharmacon and cloned into the pCMV-Sport6 vector for overexpression assays. The plasmid pcDNA3.1 mRuby was obtained from Dr. Jianbo Wu from Dr. Radbod Darabi lab.

### *Olig2* 3′ UTR ZsGreen1 reporter transfections and flow cytometry

293FT cells were transiently transfected using X-tremeGENE HP DNA Transfection Reagent (Roche) under recommended conditions. A 2.77-μg aliquot of total plasmid DNA was used in each well of 6-well plate transfections. Along with the pcDNA3.1 mRuby vector, ZsGreen1 reporter constructs including pZsGreen1-*Olig2* 3′ UTR or pZsGreen1 empty vector were co-transfected with pCMV-Sport 6-*Olig2* vector or empty vector pCMV-Sport 6 at equal molar ratios into 293FT cells in triplicate for each group. Cells were harvested forty-eight hours after transfection, and flow cytometry (BD FACSAria II) analysis was performed to get MFI of ZsGreen1 and mRuby signals. Data were analyzed using FlowJo software (FlowJo.com). The statistically significant difference between the quantified signals was analyzed with paired two-tailed Student’s *t* test (n = 6). All quantified data were presented as mean ± SEM (standard error of the mean).

### NSC transfection

3 × 10^6^ NSCs were re-suspended in 100 μl P3 primary cell solution with plasmid DNA of ZsGreen1 reporter constructs with or without *Olig2* 3′ UTR. 100 μl Nucleocuvettes were used for transfection of NSCs with the DS-112 program applied [87]. Then the cells were diluted in 500 μl warmed NSC medium, pelleted by centrifugation, then were re-suspended in NSC medium and cultured on 6 well plates (coated by Poly-L-Ornithine and Fibronectin), and incubated at 37 °C in humidified air with 5% CO_2_.

For Western blot, the whole-cell lysate of the NSCs was prepared in RIPA buffer (50 mM Tris·HCl at pH 7.4, 150 mM NaCl, 1% Triton x-100, 1% sodium deoxycholate, 0.1% SDS, 1 mM EDTA with Protease Inhibitor Mixture) and collected as supernatant after centrifugation at 10,000×*g* for 5 min. Samples were subjected to SDS-PAGE electrophoresis followed by electrotransfer to PVDF membranes. After transfer, Western blot was performed using the Pierce Fast Western Blot kit (Thermo Fisher Scientific Inc.), according to the manufacturer’s instructions [88]. Briefly, membranes were incubated with primary antibodies such as Rabbit anti-OLIG2 (1:3000) or Rabbit anti-GAPDH (1:1000) overnight at 4 °C. Bound primary antibodies were probed with horseradish-peroxidase-conjugated secondary antibodies, visualized by enhanced chemiluminescence, photographed by X-ray films, and quantified with the ImageJ software. The statistically significant difference between the quantified signals was analyzed with paired two-tailed Student’s *t* test (n = 3). All quantified data were presented as mean ± SEM (NSC+ZsGreenUTR vs NSC+ZsGreen group: 2.78 ± 1.37 vs 1.32 ± 0.18; both groups were normalized by setting NSC group as 1).

### RNA-Seq and ChIP-Seq data analysis in NSCs, OPCs, and NFOs

RNA-Seq data for eight purified brain cell types (NSCs, neurons, astrocytes, microglia, OPCs, NFOs, MOs (myelinating oligodendrocytes), and endothelial cells) are from our previous papers [21, 43]. Read mapping, transcript assembly, and expression estimation were performed in the same manner as in our previous publications [43, 89]. Reads were aligned to the mm10 reference genome using TopHat v2.1.0 [90] and FPKM (Fragments Per Kilobase of transcript per Million mapped reads) values were obtained using Cufflinks v2.2.1 [91] with default parameters. Differentially expressed genes (DEGs) were analyzed using DEseq2 [92]. The significant DEGs were filtered according to the criteria |log2 (fold-change)| > 1, FDR < 0.01, and at least one sample with FPKM > 1.

ChIP-Seq of OLIG2, H3K4me3, and H3K27me3 in NSCs, OPCs, and NFOs were performed on the Illumina platform. All reads were mapped to the mm10 reference genome using Bowtie2 [93], and only unique mapped reads were used for subsequent analysis. OLIG2 ChIP-Seq data were analyzed using the transcription factor pipeline from ENCODE (https://github.com/kundajelab/chipseq_pipeline). Peaks for OLIG2 were called using SPP and filtered to exclude regions blacklisted by ENCODE. Resulting called peaks with FDR < 0.05 and fold-enrichment > 5 were used for downstream analysis. ChIP-Seq for H3K4me3 and H3K27me3 were analyzed using MACS2 (broad peak) and filtered to exclude the ENCODE blacklist regions. Only called peaks with FDR < 0.05 and fold-enrichment > 3.5 were used for further analysis. The genome-wide distribution of OLIG2 called peaks in NSCs, OPCs, and NFOs was analyzed using ChIPseeker [94]. To obtain a comprehensive mouse lncRNA database, we combined the known and predicted lncRNA annotation from GENCODE (ftp://ftp.ebi.ac.uk/pub/databases/gencode/Gencode_mouse/release_M14/) and NCBI (ftp://ftp.ncbi.nih.gov/genomes/refseq/vertebrate_mammalian/Mus_musculus/all_assembly_versions/GCF_000001635.25_GRCm38.p5/). Annotated OLIG2 called peaks lying within 5 kb upstream of the TSS of a gene and within its CDS were retained for further analysis. Motif analysis based on the peak location within these regions was performed using HOMER motif tools (findMotifs.pl) [2]. When H3K4me3 and H3K27me3 called peaks overlapped, we merged the peak regions using “bedtools merge” into bivalent domains [95]. The H3K4me3 only peaks, H3K27me3 only peaks, and bivalent domain were annotated based on the 2 kb upstream and downstream of the TSS. The genes annotated with bivalent domains but no H3K4me3 only or H3K27me3 only peaks were designed as bivalent genes.

### RIP-Seq data analysis in NSCs

To identify potential OLIG2 binding sites we used ASPeak [96]. ASPeak is a peak detection algorithm that is sensitive to differential expression levels of target transcripts. We ran ASPeak with default parameters and used NSCs RNA-Seq data to estimate parameters *p* and *r* for the negative binomial distributions. We used NSCs IgG RIP-Seq as a control library. Gene annotations were performed with mm10 GENCODE file (release GRCm38). To determine statistically significant OLIG2 called peaks, we used three criteria: (a) tag count fold-change between OLIG2 RIP-Seq and IgG control greater than 2, (b) OLIG2 RIP-Seq *p* value less than 0.01, and (c) transcript RPKM greater than 1. Additional file 7: Table S5 includes a list of the significant OLIG2 targets found.

### Publicly accessible regulation database for oligodendrocyte development

To provide the called peaks of H3K4me3 (K4) and H3K27me3 (K27) in NSCs, OPCs, and NFOs as an accessible resource [49], we presented the peaks in this study using the WashU EpiGenome Browser, accessible at the following link: http://jiaqianwulab.org/OLIG2chip/oligodendrocyte_chip.html.

### Cell-type-specific expression lncRNAs

The cell-type-specific lncRNAs were identified using Z-scores [97]. The expression values of each gene were transformed into Z-scores. The transformed Z-score expression values were used to characterize specific expression pattern in each cell type. Genes with large positive Z-scores had particularly high expression in one cell type [97]. We sorted the Z-score expression values for the genes in each cell type and designated the top 100 lncRNAs as lncRNAs with cell-type-specific expression.

### *Olig2* overexpression RNA-Seq analysis

RNA-Seq data for *Olig2* overexpression in neural progenitors were downloaded from NCBI (GEO accession number: GSE65462). Read mapping, transcript assembly, and expression estimation were the same as the former. Three control samples (two other control samples were removed because of their low correlation with the control samples that were used) and two overexpression samples were used for subsequent analyses. DEGs were analyzed using DEseq2 [92]. Genes with significantly variable expression were filtered according to *p* value < 0.05, |fold-change| > 1.5, and at least one sample with FPKM > 1.

### Gene set enrichment analysis

We used H (hallmark gene sets), C2, and C5 curated gene sets to analyze the enriched functional GO terms and KEGG pathways [98]. A hypergeometric distribution was used to identify significant GO terms and KEGG pathways. Gene sets with an FDR < 0.05 were considered enriched.

### Predicting potential function of lncRNAs

We used a “guilt-by-association” method to infer putative functions of lncRNAs [89, 99]. Possible relationships between the expression of lncRNAs and protein coding genes were calculated using Pearson correlation. The ranked list of protein-coding genes for each lncRNA was used to identify significantly enriched gene sets corresponding to gene ontologies and canonical pathways from MSigGdb [98] using GSEA [46, 47]. Gene sets with an FDR < 0.25 were used to generate an association matrix [89]. Normalized enrichment scores (NES) were used to designate significantly enriched gene sets [89].

## Supplementary Information

**Additional file 1: Table S1.** The annotation of *Olig2* binding peaks in NSCs, OPCs, and NFOs (upstream 5 kb plus genebody).

**Additional file 2: Table S2.** The differentiation expressed genes of OPCs vs. NSCs and NFOs vs. OPCs (At least one sample's (NSCs/OPCs/NFOs) FPKM> 1, FDR < 0.01 and |log2(FoldChange)|> 1).

**Additional file 3: Table S3.** The gene expression patterns of lncRNA bound by OLIG2 in NSCs, OPCs and NFOs.

**Additional file 4: Fig S1.** The expression of *AC140285.1* in NSCs, OPCs and NFOs. The bar graphs show mean ± SEM (Standard error of the mean)

**Additional file 5: Table S4.** The annotation of ‘K4 only’, ‘K27 only’, and bivalent domains in NSCs, OPCs and NFOs (upstream and downstream 2 kb of TSS).

**Additional file 6: Fig S2.** a-c, Profiles of H3K4me3 and H3K27me3 marks and RNA-Seq are visualized using IGV (*Olig2* (a), *Yy1* (b) and *Gpr17* (c))

**Additional file 7: Table S5.** A list of statistically significant targets in OLIG2 RIP-Seq experiments

**Additional file 8: Fig S3.** Schematic representation of biotin-labeled RNA for RNA pull-down assay (a) and plasmid DNA used for transfection (b).

**Additional file 9: Fig S4.** a. Eleven Clusters of cell-type-specific lncRNAs and the expression of *Crnde* in eight brain cell types. b. ChIP-Seq profiles of *Gm28513* in NSC/OPC/NFO visualized using IGV, and expression of *Gm28513* in *Olig2*-overexpressing NPCs, NSCs and OPCs

**Additional file 10: Fig S5.** a, b. Heatmaps: 367 genes have bivalent domains in NSCs but with only H3K4me3 marks in OPCs and NFOs (a); 72 genes have bivalent domains in NSCs and OPCs but only H3K4me3 marks in NFOs (b).

**Additional file 11: Fig S6.** Interaction of *lnc-OPC* with EZH2 and SUZRIP is shown using NSCs lysate with anti-EZH2 or anti-SUZ12 as the immunoprecipitating antibody. IgG immunoprecipitation was used as a negative control. Purified RNA was then analyzed by qRT-PCR using primers specific for *lnc-OPC*. Fold-enrichment over IgG was calculated using the ΔΔCt method. Results represent three independent biological replicates; the bar graphs show means ± Standard Error. *t*-test analysis * *p* < 0.05.

**Additional file 12: Fig S7.** a, The presence of AU-rich elements in 3’ UTR of *Olig2* mRNA identified by searching AREsite2 database. b, Highly conserved *Olig2* AU-rich regions in multiple species

**Additional file 13: Fig S8.** Overview of the website.

## Data Availability

The datasets generated in the current study are available in the GEO dataset: GSE155890. The called peaks in OLIG2, H3K27me3 (K27), and H3K4me3 (K4) ChIP-Seq datasets in NSCs, OPCs, and NFOs are publicly available at http://jiaqianwulab.org/OLIG2chip/oligodendrocyte_chip.html (Additional file 13: Fig S8).

## Abbreviations

CNS: Central nervous
ESC: Embryonic stem cell
iPSCs: Inducible pluripotency cells
NSCs: Neural stem cells
NFOs: Newly formed oligodendrocytes
MOs: Myelinating oligodendrocytes
OPCs: Oligodendrocyte progenitor cells
NSPCs: Neural stem/progenitor cells
SVZ: Subventricular zone
AREs: AU-rich elements
UTR: Untranslated region
CDS: Coding sequence only
IRES: Internal Ribosome Entry Site
TSS: Transcription start sites
TF: Transcription factor
lncRNAs: Long non-coding RNAs
bHLH: Basic-helix-loop-helix
lincRNAs: Long intergenic noncoding RNAs
FPKM: Fragments Per Kilobase of transcript per Million mapped reads
DEGs: Differentially expressed genes
K27: H3K27m3
K4: H3K4m3
ChIP-Seq: Chromatin immunoprecipitation sequencing
RIP-Seq: RNA immunoprecipitation-sequencing
RNA-Seq: RNA sequencing
GSEA: Gene Set Enrichment Analysis
NES: Normalized enrichment scores
DNase-DGF: Digital DNase Genomic Footprinting
ENCODE: The Encyclopedia of DNA Elements
MFI: Median fluorescent intensity
