# Supplementary figures and images for: OLIG2 regulates lncRNAs and its own expression during oligodendrocyte lineage formation
Source: BMC Biol. 2021 Jun 25;19:132. doi: 10.1186/s12915-021-01057-6 (PMC8235854; doi:10.1186/s12915-021-01057-6)

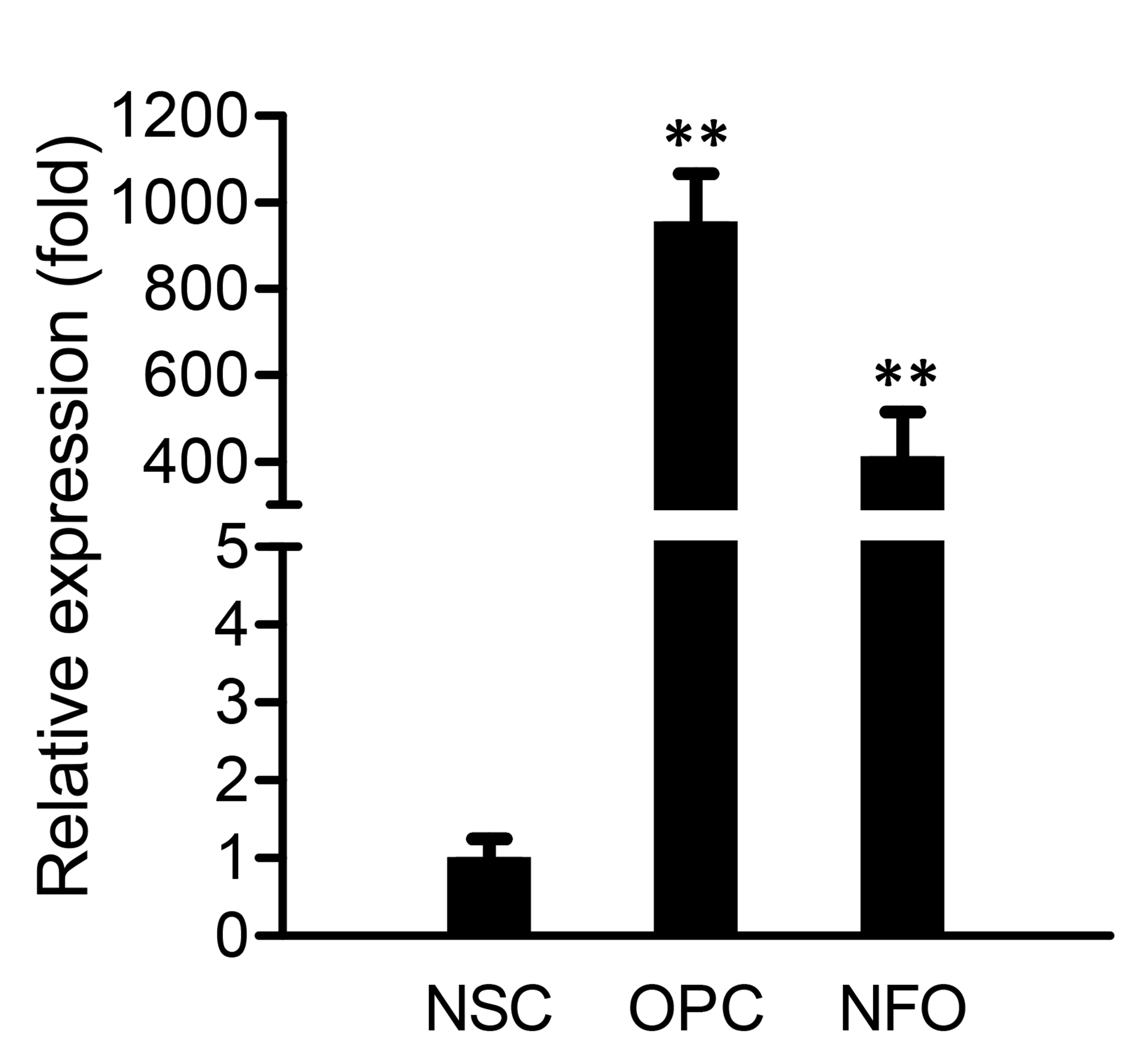

Supplement: Supplementary file 4 — Additional file 4: Fig S1. The expression of AC140285.1 in NSCs, OPCs and NFOs. The bar graphs show mean ± SEM (Standard error of the mean) [file 12915_2021_1057_MOESM4_ESM.tif]

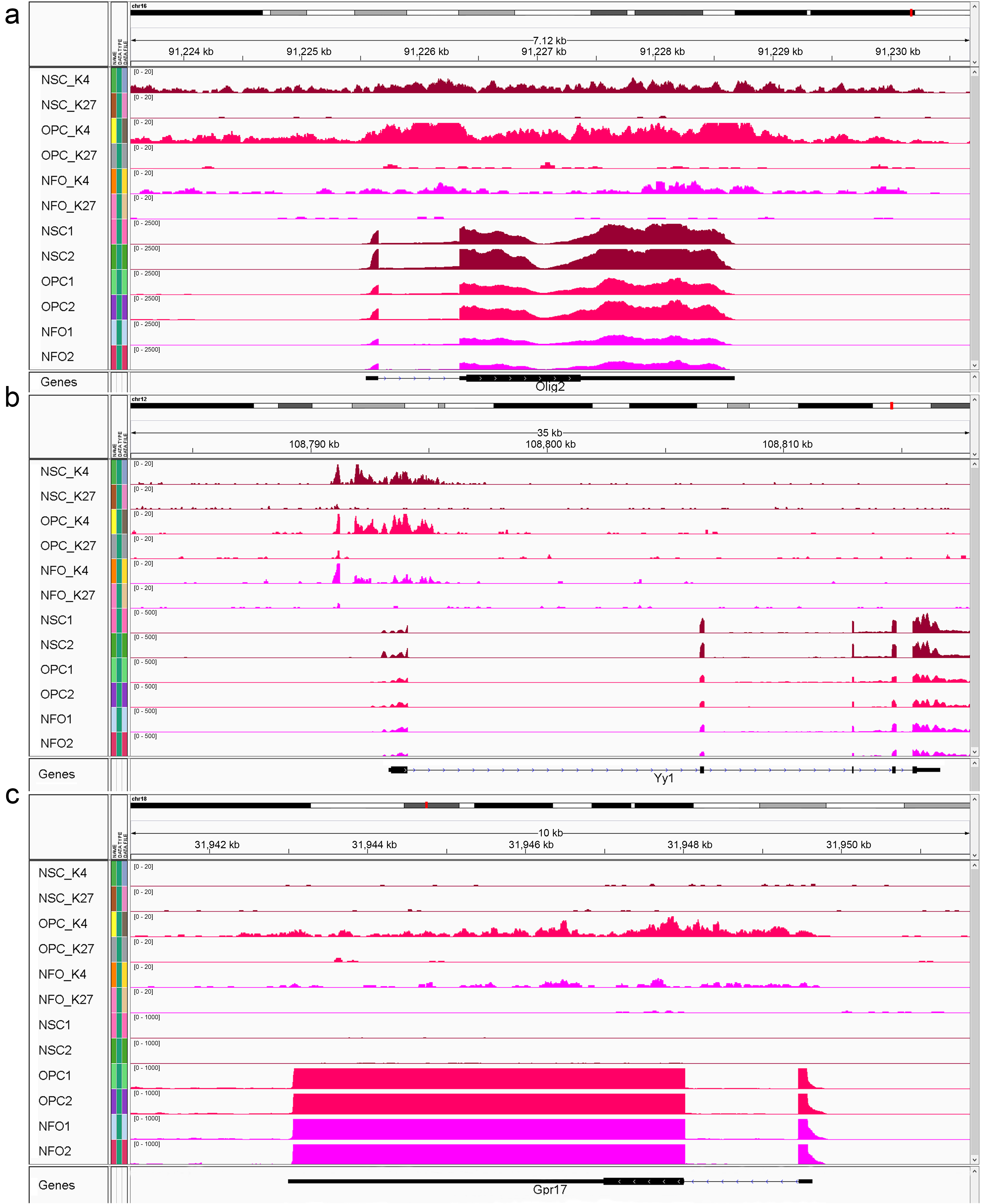

Supplement: Supplementary file 6 — Additional file 6: Fig S2. a-c, Profiles of H3K4me3 and H3K27me3 marks and RNA-Seq are visualized using IGV (Olig2 (a), Yy1 (b) and Gpr17 (c)) [file 12915_2021_1057_MOESM6_ESM.tif]

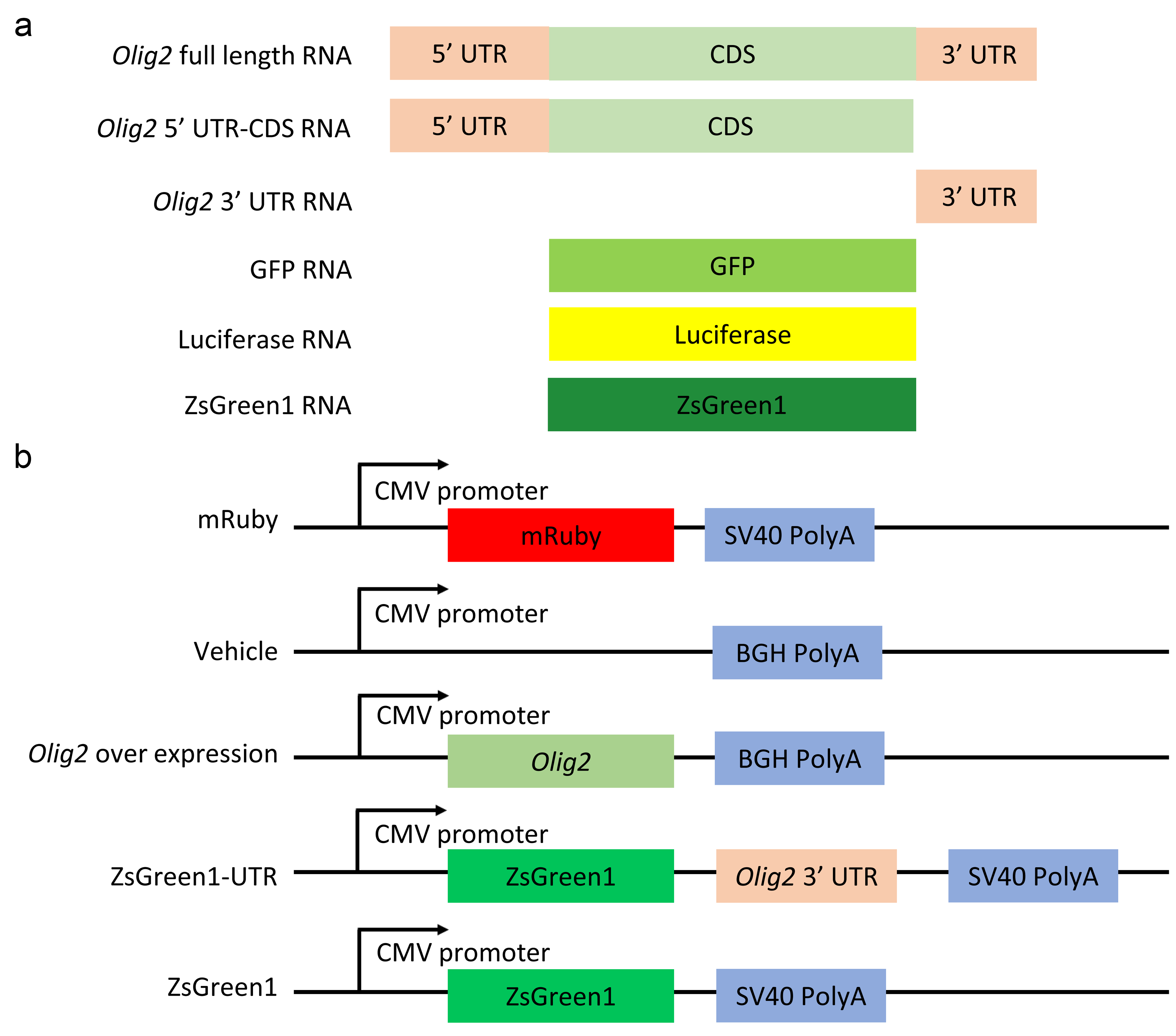

Supplement: Supplementary file 8 — Additional file 8: Fig S3. Schematic representation of biotin-labeled RNA for RNA pull-down assay (a) and plasmid DNA used for transfection (b). [file 12915_2021_1057_MOESM8_ESM.tif]

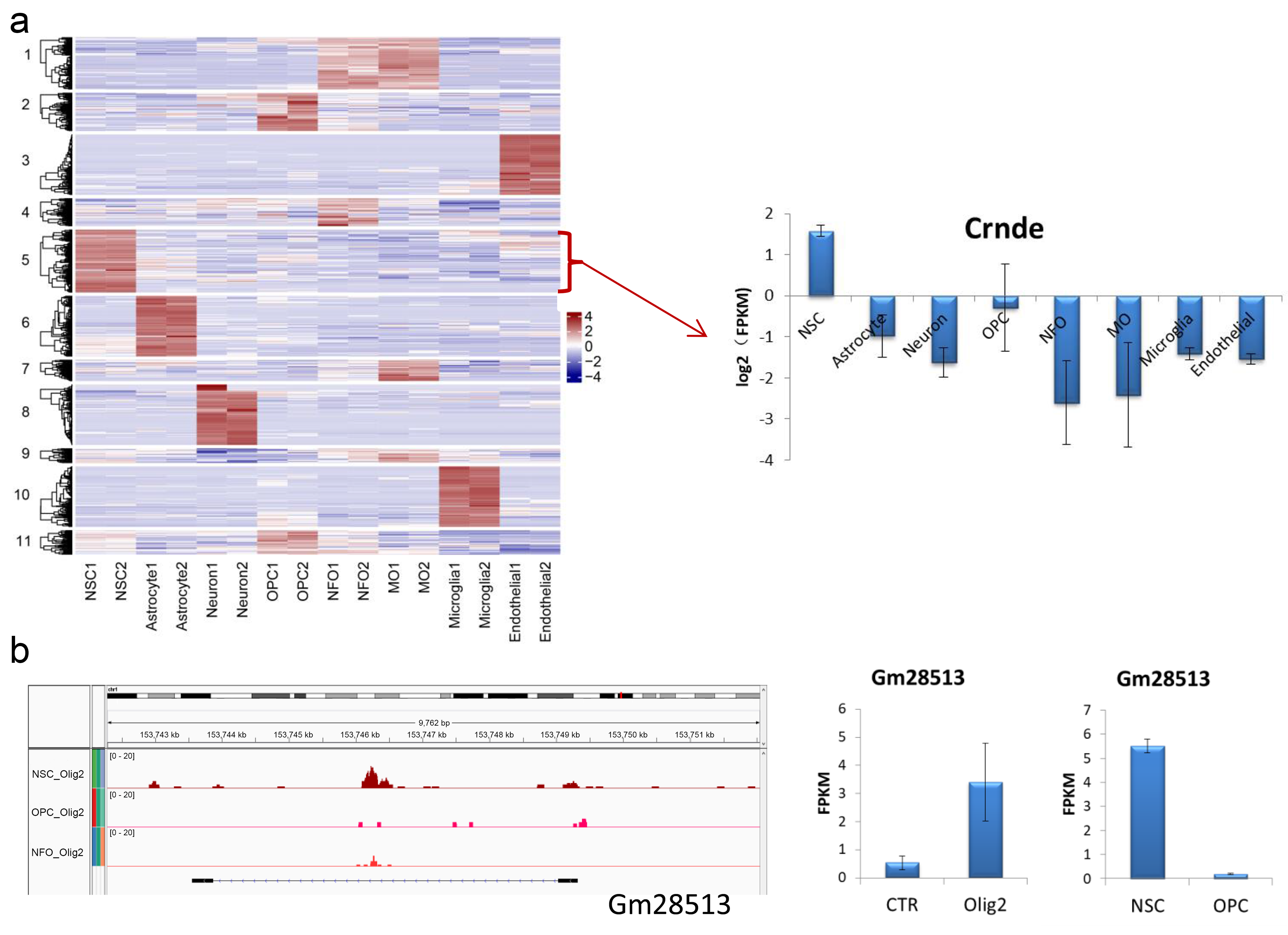

Supplement: Supplementary file 9 — Additional file 9: Fig S4. a. Eleven Clusters of cell-type-specific lncRNAs and the expression of Crnde in eight brain cell types. b. ChIP-Seq profiles of Gm28513 in NSC/OPC/NFO visualized using IGV, and expression of Gm28513 in Olig2-overexpressing NPCs, NSCs and OPCs [file 12915_2021_1057_MOESM9_ESM.tif]

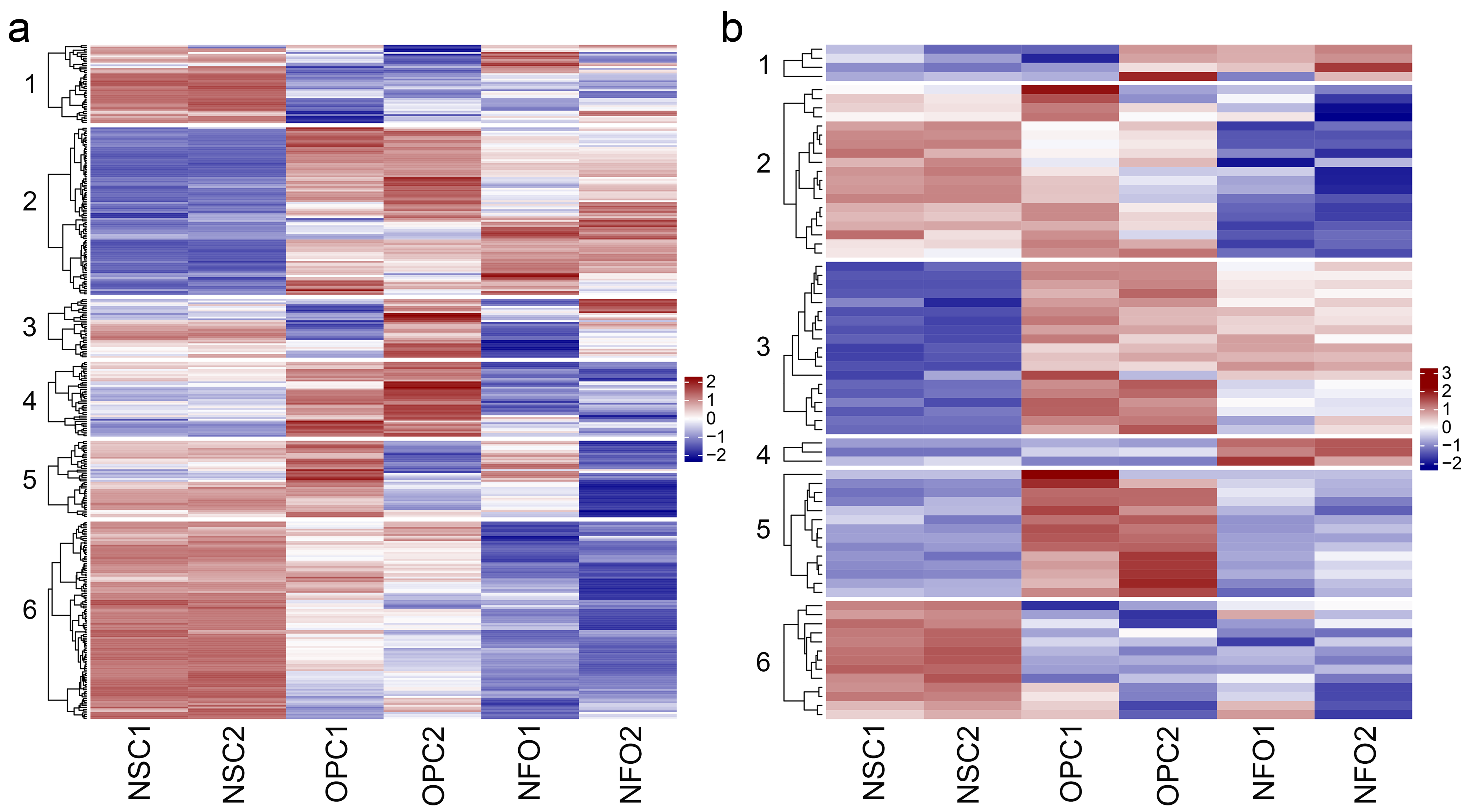

Supplement: Supplementary file 10 — Additional file 10: Fig S5. a, b. Heatmaps: 367 genes have bivalent domains in NSCs but with only H3K4me3 marks in OPCs and NFOs (a); 72 genes have bivalent domains in NSCs and OPCs but only H3K4me3 marks in NFOs (b). [file 12915_2021_1057_MOESM10_ESM.tif]

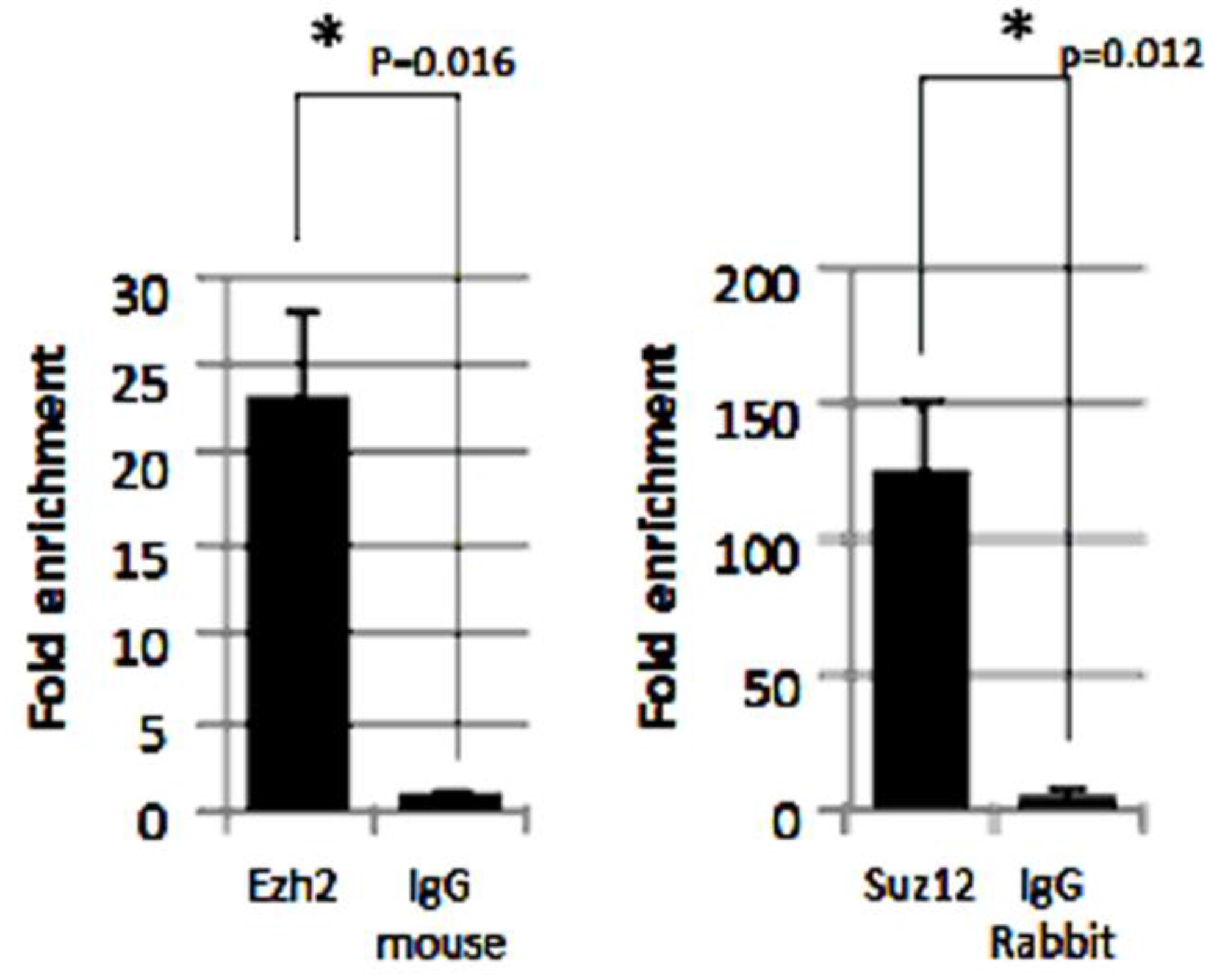

Supplement: Supplementary file 11 — Additional file 11: Fig S6. Interaction of lnc-OPC with EZH2 and SUZRIP is shown using NSCs lysate with anti-EZH2 or anti-SUZ12 as the immunoprecipitating antibody. IgG immunoprecipitation was used as a negative control. Purified RNA was then analyzed by qRT-PCR using primers specific for lnc-OPC. Fold-enrichment over IgG was calculated using the ΔΔCt method. Results represent three independent biological replicates; the bar graphs show means ± Standard Error. t-test analysis * p < 0.05. [file 12915_2021_1057_MOESM11_ESM.tif]

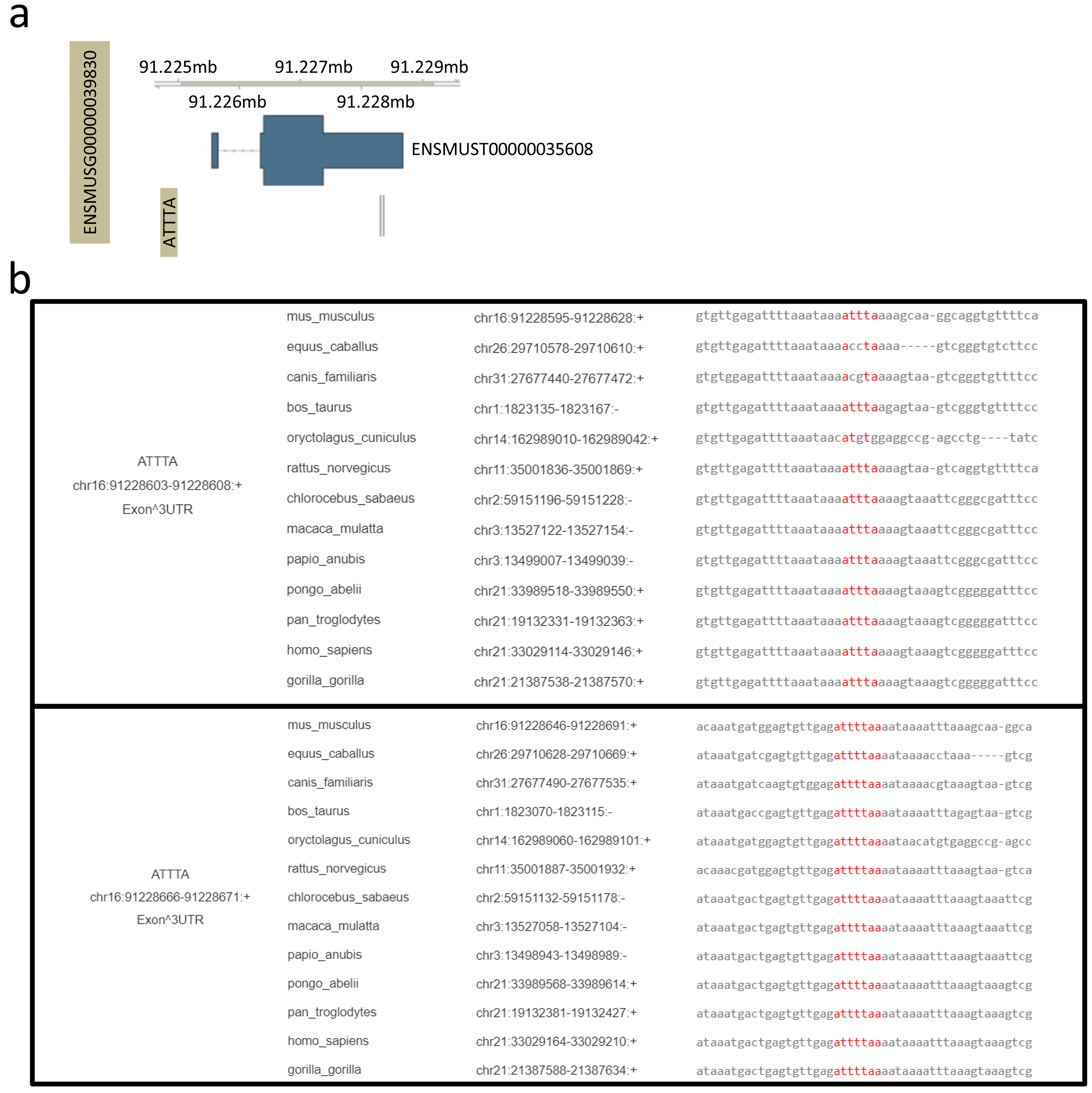

Supplement: Supplementary file 12 — Additional file 12: Fig S7. a, The presence of AU-rich elements in 3’ UTR of Olig2 mRNA identified by searching AREsite2 database. b, Highly conserved Olig2 AU-rich regions in multiple species [file 12915_2021_1057_MOESM12_ESM.tif]

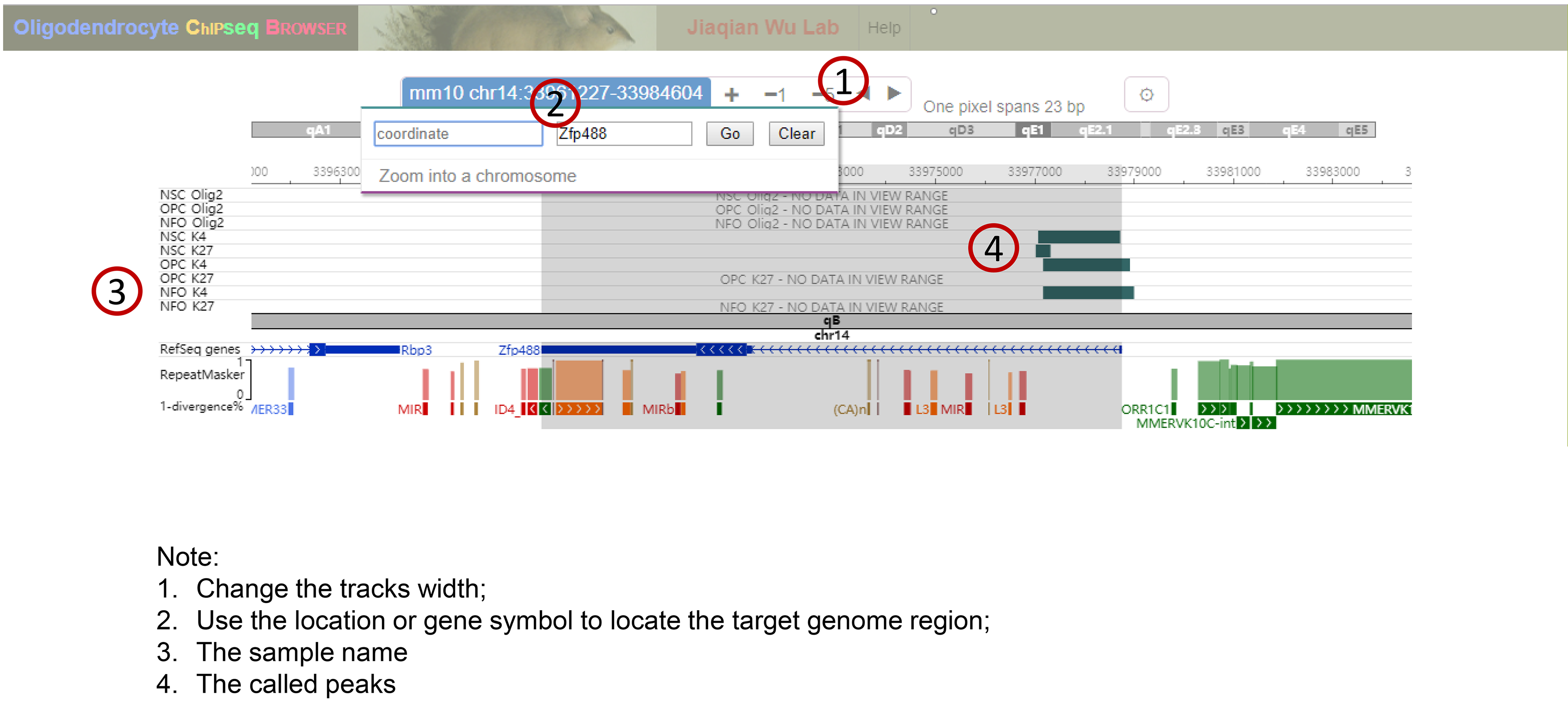

Supplement: Supplementary file 13 — Additional file 13: Fig S8. Overview of the website. [file 12915_2021_1057_MOESM13_ESM.tif]
